# Systems biology surveillance decrypts pathological transcriptome remodeling

**DOI:** 10.1186/s12918-015-0177-8

**Published:** 2015-07-17

**Authors:** Randolph S. Faustino, Saranya P. Wyles, Jody Groenendyk, Marek Michalak, Andre Terzic, Carmen Perez-Terzic

**Affiliations:** Division of Cardiovascular Diseases, Departments of Medicine, Molecular Pharmacology and Experimental Therapeutics, Mayo Clinic, 200 First Street SW, Rochester, MN 55905 USA; Department of Biochemistry, University of Alberta, Edmonton, AB Canada; Department of Physical Medicine and Rehabilitation, Mayo Clinic College of Medicine, Rochester, MN USA; Rehabilitation Medicine Research Center, Rochester, MN USA

**Keywords:** Stem cells, Pluripotent, Microarray, Gene ontology, Network, Transcriptome, Bioinformatics, Systems biology

## Abstract

**Background:**

Pathological cardiac development is precipitated by dysregulation of calreticulin, an endoplasmic reticulum (ER)-resident calcium binding chaperone and critical contributor to cardiogenesis and embryonic viability. However, pleiotropic phenotype derangements induced by calreticulin deficiency challenge the identification of specific downstream transcriptome elements that direct proper cardiac formation. Here, differential transcriptome navigation was used to diagnose high priority calreticulin domain-specific gene expression changes and decrypt complex cardiac-specific molecular responses elicited by discrete functional regions of calreticulin.

**Methods:**

Wild type (WT), calreticulin-deficient (CALR^−/−^), and calreticulin truncation variant (CALR^−/−^-NP and CALR^−/−^-PC) pluripotent stem cells were used to investigate molecular remodeling underlying a model of cardiopathology. Bioinformatic deconvolution of isolated transcriptomes was performed to identify predominant expression trends, gene ontology prioritizations, and molecular network features characteristic of discrete cell types.

**Results:**

Stem cell lines with wild type (WT), calreticulin-deficient (CALR^−/−^) genomes, as well as calreticulin truncation variants exclusively expressing either the chaperoning (CALR^−/−^-NP) or the calcium binding (CALR^−/−^-PC) domain exhibited characteristic molecular signatures determined by unsupervised agglomerative clustering. Kohonen mapping of RNA expression changes identified transcriptome dynamics that segregated into 12 discrete gene expression meta-profiles which were enriched for regulation of Eukaryotic Initiation Factor 2 (EIF2) signaling. Focused examination of domain-specific gene ontology remodeling revealed a general enrichment of Cardiovascular Development in the truncation variants, with unique prioritization of “Cardiovascular Disease” exclusive to the cohort of down regulated genes of the PC truncation variant. Molecular cartography of genes that comprised this cardiopathological category revealed uncharacterized and novel gene relationships, with identification of *Pitx2* as a critical hub within the topology of a CALR^−/−^ compromised network.

**Conclusions:**

Diagnostic surveillance, through an algorithm that integrates pluripotent stem cell transcriptomes with advanced high throughput assays and computational bioinformatics, revealed collective gene expression network changes that underlie differential phenotype development. Stem cell transcriptomes provide a deep collective molecular index that reflects *ad hoc* robustness of the pluripotent gene network. Remodeling events such as monogenic lesions provide a background by which high priority candidate disease effectors and regulators can be identified, demonstrated here by a molecular profiling algorithm that decrypts pluripotent wild type versus disrupted genomes.

**Electronic supplementary material:**

The online version of this article (doi:10.1186/s12918-015-0177-8) contains supplementary material, which is available to authorized users.

## Background

A functional myocardium is the culmination of intricate dynamic signaling orchestrated by regulated processes [1, 2], and disruptions of cardiogenesis that lead to congenital cardiac defects occur in as high as 8 % of prenatal cases [3, 4]. Discrete molecular elements that give rise to inborn cardiac errors are numerous and precipitate developmental cardiopathology through multiple mechanisms including inhibition of cardiomyocyte proliferation and differentiation [5], abrogation of syncytial/electrical function [6], and disruption of nascent myocardial anatomy [7, 8]. Precise models of cardiac development that report phenotype derangement specific for a targeted gene have enhanced resolution of signaling axes that drive clinical pathology, yet these platforms are of limited use for anticipatory diagnosis of cardiopathology in advance of symptomatic presentation. Present methods to detect congenital heart defects are limited to time frames proximal to phenotype onset, which critically restricts opportunities for curative or palliative interventions.

Pre-manifestation *in silico* extrapolation of cardiac defects is feasible with the use of large volume, multidimensional biodata, as high throughput techniques generate profound datasets which could serve as the foundation for incisive dissection of complex molecular patterns [9]. Indeed, continuous development of bioinformatic tools expands the range of quantifiable properties of “omic” data, and provides the basis for advanced systems biology algorithms of deconvolution [10]. Resolution of the intricate molecular framework underlying phenotype dynamics using this interdisciplinary methodology has been successful [9, 11], yet parsing diverse and complex datasets remains an extant challenge.

Embryonic stem (ES) cells harbor a pluripotent genome with an engaged pre-differentiation molecular landscape [12, 13], and the naive transcriptome of an ES cell provides a unique source of material for exploration of the primordial gene network that supports normal development [14–17]. Disruptions within this principal transcriptome may ultimately drive pathological onset and disease progression, thus elucidation of primary transcriptome dynamics under normal and diseased conditions is required for advanced refinement of disease prediction [9, 18]. Case in point, ES cells that lack a functional calreticulin gene, i.e. *calr*^*−/−*^, abrogates calreticulin (CALR) expression, and provides a robust model for interrogation of a discrete mechanism of pathological cardiogenesis [19, 20]. Systems biology dissection of prioritized gene ontology remodeling in *calr*^*−/−*^ ES cell lines revealed the underlying molecular network that drove ultrastructural, functional, and anatomical derangements in cardiac phenotype [18]. However, CALR is a Ca^2+^-handling chaperone [21, 22], and specific contributions of its particular functional domains, i.e. calcium binding *versus* protein folding, to final phenotype has remained uncharacterized.

Dissection of the potential contributions of each CALR domain to global phenotype generation would identify differential gene ontology remodeling specified by distinct regions within a multifunctional protein. This would refine and increase resolution of the primordial CALR-driven molecular atlas preceding organogenesis. Here, using an algorithm of bioinformatic deconvolution to unravel the intricate transcriptome networks harbored by calreticulin truncation variants, the contributions of the protein chaperoning domain (NP) *versus* the calcium handling (PC) region were investigated. Truncation variants, generated by transfection of CALR^−/−^ cell lines with constructs that exclusively expressed the protein chaperoning (CALR^−/−^-NP) or the calcium handling (CALR^−/−^-PC) domain, were used to determine differential Gene Ontology enrichments coded for by each CALR functional region. To build upon previous work, dysfunctional transcriptome recalibration preceding cardiogenesis was prioritized. The classification of Cardiovascular Development was enriched for by both truncation variants, though the down regulated cohort of genes in the transcriptome of the CALR^−/−^-PC truncation variant exhibited specific prioritization of genes that promote Cardiovascular Disease. To determine functional interconnectivity of genes within this pathological category, molecular cartography was used to reveal a local scale-free network with significant prioritization of genes previously uncharacterized as part of CALR-governed cardiogenesis. Advanced systems biology-derived algorithms, combined with the molecular dynamics of pluripotent ES cells, thus facilitates decryption of covert gene network features and functional enrichment remodeling actuated by discrete regions within CALR that drive phenotype manifestation.

## Results

### Dataset generation and global transcriptome profiling

**Fig. 1:**
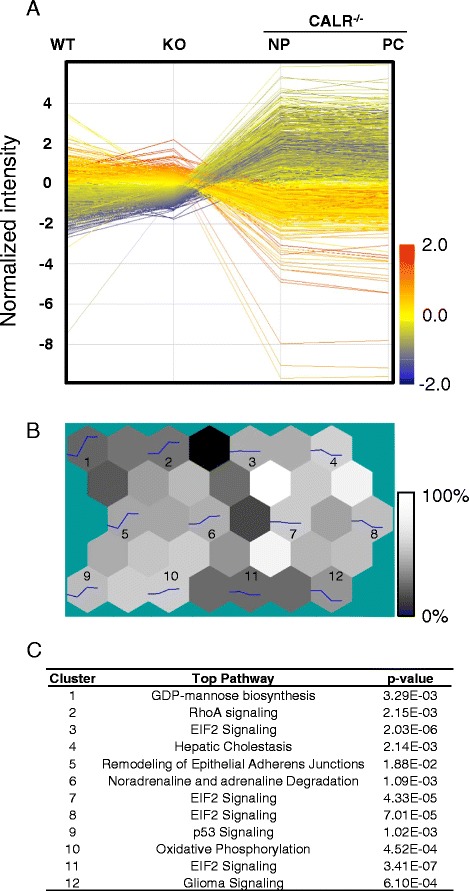
Profile expression deconvolution. **a** Background normalized transcripts according to P, M, and A flag values streamlined Affymetrix probeset populations that were further refined to report transcripts with significant expression changes (*P* <0.01). The resulting transcript dynamics were profiled to examine magnitude and direction of expression changes, which revealed diverse gene expression patterns. **b** Kohonen mapping, or Self-Organizing Maps (SOM), categorized the transcriptome into 12 discrete clusters, visualized as a U-matrix. Multidimensional data visualized by U-matrices are organized into hexagon plots of alternating nodes (containing gene cluster metaprofiles) and non-nodes (indicating similarity). Each node is numbered and presents the metagene expression profile for that cluster, while non-nodes indicate percent similarity between clusters according to Euclidean distance metrics. Grayscale bar indicates high (white) to low (black) percent similarity. **c** Top pathways prioritized for each cluster, with *p*-value indicated for each

Gene expression changes among wild type (WT), CALR deficient (*calr*^*−/−*^) and CALR domain-specific truncation variant cell lines were observed for 5034 quality controlled expression profiles (Fig. 1a). Bioinformatic mining of rich profile data via hierarchical clustering revealed that transcriptome footprints could be segregated into 12 discrete expression trends according to mean profile dynamics via Kohonen mapping (Fig. 1b). Prioritized signaling pathways for each cluster exhibited specific functional enrichment upon analysis by Ingenuity Pathways Analysis (Fig. 1c). Significantly, Eukaryotic Initiation Factor-2 (EIF2) Signaling was the top pathway for Clusters 3, 7, 8, and 11, with many ribosomal proteins within this pathway differentially regulated among the four conditions (Additional file 1). Indeed, at this level of analysis, enrichment in general categories of Cellular Growth, Cellular Development, and Cellular Organization were among the top networks for each cluster (Additional file 1). Broad transcriptome deconvolution thus provides a definable overview of enriched functional themes independent of genomic context.

### Domain-specific transcriptome dissection and gene ontology enrichment

**Fig. 2:**
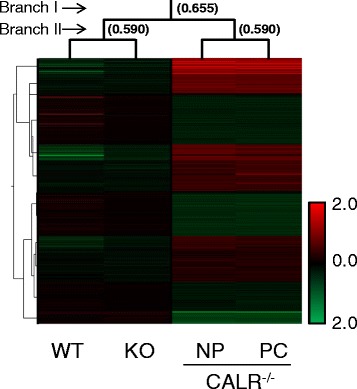
Transcriptome signatures of ES cell lines. Gene expression profiling of global mRNA levels from WT (*n* = 2), *calr* ^*−/−*^(*n* = 5), NP (*n* = 3) and PC (*n* = 3) truncation variants were analyzed by agglomerative unsupervised clustering analysis, with mean expression of genes in each condition organized into a heatmap dendrogram. Data represents an update of GEO dataset (GDS3729). WT and *calr* ^*−/−*^ together were distinct from NP and PC profiles, with a calculated Euclidean distance metric of 0.655 (Branch I). Distance metrics between WT and *calr* ^*−/−*^ and between NP and PC variants indicate a similar degree of distinction at Branch II. Colorscale indicates normalized intensity values as a metric of expression for up and down regulated genes in red and green, respectively

Previous transcriptome analysis of WT and *calr*^*−/−*^ cell lines identified gene expression network remodeling underlying cardiac phenotype derangement in the knockout condition [18]. However, CALR is a multifunctional protein with chaperoning and calcium binding functions, and its effects on systems wide transcriptome dynamics may be differentially regulated by either or both of its protein domains. Transcriptomes from WT, *calr*^*−/−*^, CALR^−/−^-NP and CALR^−/−^-PC cell lines were profiled by microarray analysis. Unsupervised agglomerative clustering by Euclidean distance revealed distinct molecular signatures for each transcriptome (Fig. 2). WT and knockout conditions, though distinct from each other, were segregated from both truncation transcriptomes with a distance metric of 0.655, whereas distance between WT and *calr*^*−/−*^, and between NP and PC, were similar, with a Euclidean distance of 0.590. Thus, transcriptome signatures of NP and PC lines could be distinguished from those of WT and knockout stem cell lines.

To investigate functional themes recruited by these specialized regions of *calr*, focused examination of NP and PC truncation variant cell lines *versus* wild type counterparts was performed. The contribution of the NP and PC variants, which exclusively express the chaperoning or calcium binding domains, respectively, may differentially contribute unique gene ontology composition to overall molecular signature. Pairwise comparison of truncation mutants to wild type cells was performed by volcano plot analysis to streamline gene profiles to transcripts changing by more than 2-fold when compared to WT, and these lists were analyzed by Ingenuity to extract significant functional annotations and gene ontology enrichments.

**Fig. 3:**
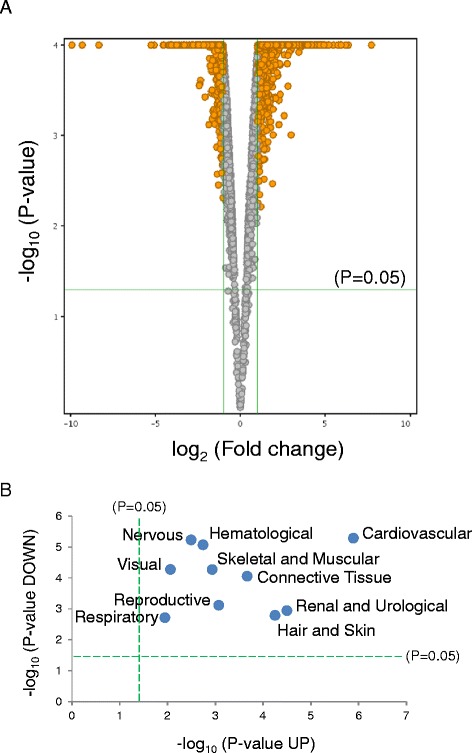
Physiological systems impacted by CALR^−/−^-NP. Quality control of the CALR^−/−^-NP transcriptome revealed enrichment of specific physiological systems. **a** Volcano plot analysis identifies genes up or down regulated by more than 2-fold *versus* control. Orange circles represent genes that meet or exceed the ±2 fold-change criteria (vertical green lines) and satisfy the *P*-value threshold of P ≤ 0.05 (horizontal green line indicates *P* = 0.05). **b** Significance plot using –log (*P*-value) reveals Cardiovascular System Development and Function as the most significantly represented developmental system impacted by NP truncation independent of up and down regulated sub-transcriptome cohorts

A total of 2936 differentially expressed genes were identified in the quality filtered CALR^−/−^-NP transcriptome (Fig. 3a). Of these, 1833 and 1103 transcripts were respectively up and down regulated when compared to expression levels in WT. Ingenuity Pathways Analysis identified significant categories of physiological development represented in both up and down regulated sub-transcriptomes (Fig. 3b and Additional file 2: Table S1). Cardiovascular System Development and Function emerged as the most significantly represented physiological category enriched within the NP transcriptome, independent of expression trend. A number of physiological categories were enriched, which included: Renal & Urological Development, Hair & Skin Development, Connective Tissue Development, Reproductive System Development, Skeletal & Muscular System Development, Hematological System Development, Nervous System Development, Visual System Development, and Respiratory System Development. In addition, enrichment of developmental systems particular to up and down regulated gene cohorts was discovered. Hepatic System Development (*P* = 4.60 × 10^−3^) was uniquely represented in up regulated genes, while categories for Endocrine (*P* = 2.13 × 10^−4^), Digestive (*P* = 9.03 × 10^−4^), and Auditory & Vestibular (*P* = 2.69 × 10^−3^) development were exclusive to the down regulated NP transcriptome complement.

Further bioinformatic mining revealed network categories and molecular functions enriched by the NP truncation mutant that included Hematological Diseases as the most enriched network function (Table 1). Specific and discrete molecular themes were prioritized for up and down regulated networks, with Cell Death and Survival, and Cellular Development among the top categories for up regulated mRNA expression changes (Table 1). In the down regulated cohort, Organismal Injury and Abnormalities and Cellular Compromise were enriched themes in the highest prioritized network (Table 1). Overall, the top specific disease profiles emerging from the NP transcriptome, inclusive of up and down regulated changes, were Cancer and Gastrointestinal Disease (Table 1).

**Table 1:** Categorical enrichment of network functions, disease classifications and/or dysregulated biological themes in the NP truncation variant

Up regulated	Down regulated
*Top Networks*	
Hematological Disease, Cell Death and Survival, Cellular Development	Hematological Disease, Organismal Injury and Abnormalities, Cellular Compromise
Cellular Development, Hematological System Development and Function, Hematopoiesis	Cellular Compromise, DNA Replication, Recombination and Repair, Cell Death and Survival
RNA Post-Transcriptional Modification, Molecular Transport, RNA Trafficking	Cellular Assembly and Organization, Cellular Function and Maintenance, Endocrine System Development and Function
Cellular Assembly and Organization, Molecular Transport, RNA Trafficking	Organismal Development, DNA Replication, Recombination and Repair, Reproductive System Development and Function
Lipid Metabolism, Small Molecule Biochemistry, Embryonic Development	Cellular Movement, Immune Cell Trafficking, Cellular Growth and Proliferation
*Top Diseases and Bio Functions*	
Cancer	Cancer
Gastrointestinal Disease	Organismal Injury and Abnormalities
Infectious Disease	Inflammatory Response
Reproductive System Disease	Developmental Disorder
Neurological Disease	Gastrointestinal Disease

**Fig. 4:**
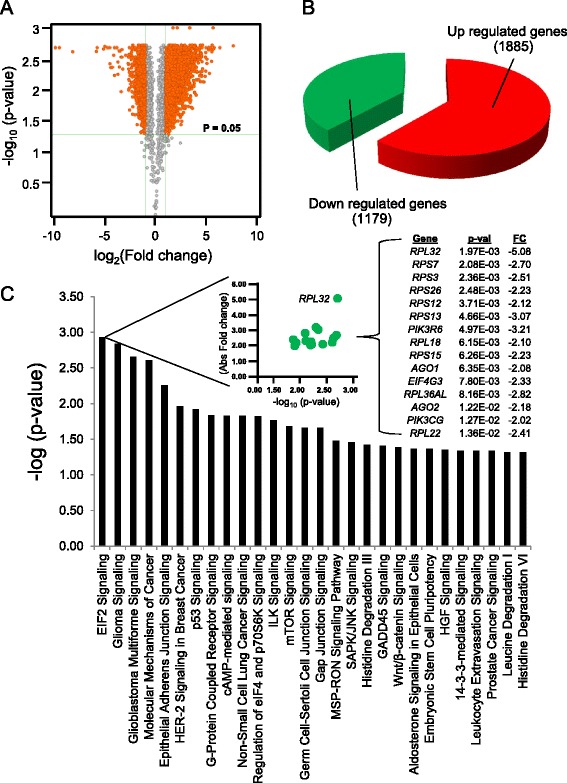
Deconvolution of cardiopathologic gene ontology remodeling discretely prioritized by CALR^−/−^-PC. Refinement of quality controlled gene lists allowed gene ontology analysis for identification of prioritized functional themes. **a** Volcano plot analysis yields up and down regulated genes that satisfy significance and fold change criteria that report specific gene ontology categories upon pathway analysis. Shown is the gene expression distribution within the quality controlled transcriptome of the PC truncation variant. Horizontal green line indicates *P*-value cutoff (*P* = 0.05). Vertical green lines delimit 2.0x fold change threshold. Orange indicates genes that meet or exceed *p*-value and fold change criteria, while gray represents genes that fall below significance and fold change limits. **b** Enriched functional themes are encoded within a finite number of up and down regulated transcripts. In the PC variant, a larger proportion of genes are up regulated. **c** As the down regulated component of the PC transcriptome harbored over representation of the Cardiovascular Disease functional category, top pathways in the down regulated component of the PC transcriptome were examined to determine identities of enriched signaling cascades. Here, EIF2 signaling was highly prioritized. Pathway nomenclature is shown on the x-axis of the histogram, while –log (*p*-value) is provided on the y-axis. *Inset* – Investigation of EIF2 signaling revealed significant down regulated expression of 15 genes, of which *RPL32* was the most significantly decreased. Shown are -log_10_ (p-value) on the x-axis, and absolute fold change on the y-axis

Interrogation of developmental ontology enrichment in the PC line also revealed prioritization of Cardiovascular System Development and Function (Additional files 3: Figure S1). However, the PC truncation variant genome elevated functional themes distinct from those observed in the NP-remodeled transcriptome. Indeed, volcano plot analysis of PC cells revealed a larger proportion of up regulated genes (Fig. 4a and b). Enrichment of Cardiovascular System Development category was preserved as the predominant physiological system coded for by up regulated transcripts in the PC transcriptome (Additional files 4: Table S2). Cellular Assembly, Molecular Transport, and RNA Trafficking are prioritized in up regulated transcripts, while Cellular Assembly and Organization, Cellular Function and Maintenance, and Hematological System Development and Function were significant categories in down regulated genes (Table 2 and Additional file 1). Similar to pathological categories revealed for the NP variant, Cancer and Gastrointestinal Disease were enriched for up regulated genes in the PC line. Within down regulated PC genes, Cancer scored highest, while the top functional organogenic category enriched for PC down regulated mRNA was Cardiovascular Disease (Table 2). Enrichment of cardiopathologic specificity within down regulated PC genes prompted closer examination of these down regulated transcripts, which revealed significant overrepresentation of 28 discrete signaling cascades, with EIF2 Signaling as the most prioritized signaling pathway (Fig. 4c).

**Table 2:** Categorical enrichment of network functions, disease classifications and/or dysregulated biological themes in the PC truncation variant

Up regulated	Down regulated
*Top Networks*	
Cellular Assembly and Organization, Molecular Transport, RNA Trafficking	Cellular Assembly and Organization, Cellular Function and Maintenance, Hematological System Development and Function
RNA Post-Transcriptional Modification, Embryonic Development, Organismal Development	Cell Signaling, Cellular Assembly and Organization, DNA Replication, Recombination, and Repair
Cell Cycle, Cellular Development, Cell Morphology	Lipid Metabolism, Molecular Transport, Small Molecule Biochemistry
Cellular Function and Maintenance, Cellular Movement, Nervous System Development and Function	Cell Death and Survival, Hematological System Development and Function, Immune Cell Trafficking
Nervous System Development and Function, Cell Cycle, Digestive System Development and Function	Cell Morphology, Nervous System Development and Function, Cell-to-cell Signaling and Interaction
*Top Diseases and Bio Functions*	
Cancer	Cancer
Gastrointestinal Disease	Cardiovascular Disease^a^
Infectious Disease	Organismal Injury and Abnormalities
Neurological Disease	Dermatological Diseases and Conditions
Developmental Disorder	Developmental Disorder

^a^The down regulated subset of genes within the PC transcriptome was the only transcript cohort that enriched for Cardiovascular Disease

### Network cartography, topological deconvolution, and signature conservation

**Fig. 5:**
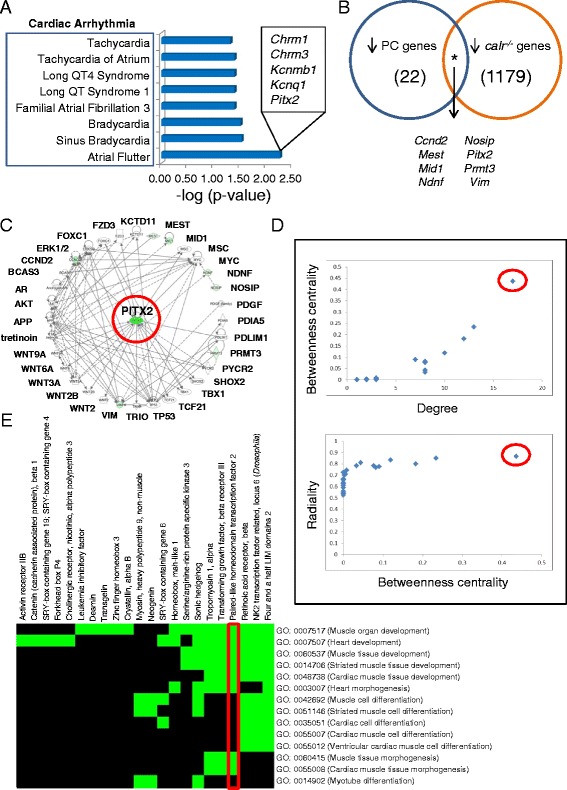
Investigation of molecular substrates that precipitate phenotypes inflicted by *calr* deficiency using bioinformatic prioritization algorithms. **a** Down regulated genes within the PC truncation variant was the only transcript expression cohort that enriched for Cardiovascular Disease. Here, focused bioinformatic extraction of Cardiovascular Disease prioritized the subcategory of Cardiac Arrhythmia, which revealed enrichment of Atrial Flutter (see text). Arrhythmogenic category shown on y-axis, with –log (*p*-value) on x-axis. *Inset –* Atrial Flutter was composed of several genes, as shown. **b** Venn diagram intersection of total down regulated genes within the PC variant (blue circle) with those down regulated in the *calr* knockout condition (orange circle) revealed eight genes consistently decreased in both conditions (see text). Numbers in parentheses indicate total number of down regulated genes for that cohort. **c** Radial layout of the network containing the eight consistently down regulated genes reveals the most connected gene as *Pitx2* (red circle). **d** Topological analysis confirms gene prioritization. *Upper panel –* plotting degree (number of connections per gene) *versus* betweenness centrality (an indicator of informational load and flow) reveals *Pitx2* as the highest priority hub (red circle). *Lower panel –* alternative plot using betweenness centrality *versus* radiality (another centrality metric that indicates node accessibility) again demonstrates prioritization of *Pitx2* (red circle). **e** Transcriptome meta-analysis by DAVID demonstrates enrichment of a cardiac cluster containing *Pitx2* (red box)

Overt prioritization of Cardiovascular Disease (*p*-val = 3.08 × 10^−5^) within the cohort of down regulated PC transcripts prompted further investigation. Streamlined bioinformatic analysis revealed a total of 54 genes that comprised the Cardiovascular Disease category (Additional file 5: Table S3). Furthermore, the top cardiac disease was identified as Cardiac Arrhythmia, and within this enriched sub-category, Atrial Flutter (*p*-val = 5.43 × 10^−3^) was identified as the most significant disease condition encoded by down regulated genes in PC cells (Fig. 5a). Specific genes within the designation of Atrial Flutter that demonstrated significant expression changes in PC cells versus wild type were *Chrm1, Chrm3, Kcnmb1, Kcnq1,* and *Pitx2*.

As an enriched cardiopathologic functional theme was uniquely and discretely identified in down regulated genes of the PC truncation line, the contribution of down regulated transcripts within the original *calr*-deficient phenotype was selected for investigation. Venn diagram intersection of down regulated PC genes with all down regulated transcripts in *calr*^*−/−*^ cells revealed common decreased expression of *Ccnd2, Mest, Mid1, Ndnf, Nosip, Pitx2, Prmt3*, and *Vim* in both transcriptomes (Fig. 5b). Network cartography of these genes revealed a streamlined 31 node sub-network (Fig. 5c). Topological analysis identified features typical of a scale-free biological network [23], where *Pitx2* possessed the highest degree, or number of connections to other nodes in the network (Fig. 5c and d).

Transcriptome meta-analysis uncovers precise molecular signatures [24], and DAVID was employed to investigate conservation of gene ontology categories identified by Ingenuity Pathways Analysis. Heatmaps of transcripts down regulated in PC *versus* wild type revealed a functional cluster that contained *Pitx2* (Fig. 5e and Additional file 1), corroborating its priority as an identified network hub.

## Discussion

Anticipatory diagnosis of disease prior to symptomatic presentation is complicated by gene expression diversity that underlies pathology. To address this, stem cell transcriptomes were utilized as *ad hoc* multidimensional indices to interrogate molecular statuses of calreticulin-disrupted cell types, which identified novel molecular dynamics that enforced cardiopathological phenotype. Identification of prioritized gene expression network hubs and altered prioritization of global molecular functions reveals intricate systems biology phenomena that comprise pathological genome remodeling, critical for advanced understanding of disease etiology.

Snapshot overviews of mRNA expression changes that underlie cellular phenotypes define transcript expression differences, gene ontology re-organization, and gene network remodeling events that can be mapped *in silico* to extract novel molecular information [9]. Here, a broad bioinformatics overview identified multiple riboproteins within Eukaryotic Initiation Factor 2 (EIF2) Signaling as priority-regulated in gene lesion variant transcriptomes. This supports reported prevalence of altered translational machinery in CALR-disrupted transcriptomes and phenotypes [18]. CALR deletion leads to impaired system wide quality control of newly synthesized glycoproteins, as well as global accumulation of conformationally compromised substrates and general abrogation of calcium-dependent protein folding processes [25–27]. Accumulation of unfolded or misfolded proteins is salvaged in part by the unfolded protein response (UPR) of the endoplasmic reticulum [28], which enacts translational re-calibration in the event of stress stimuli. As part of this response, EIF pathways are targeted to facilitate restoration of protein homeostasis through prevention of misfolded protein accumulation that occurs under conditions of physiological and/or environmental duress, while concomitantly stimulating cells that contain conformationally defective proteins to initiate apoptosis [29–32]. Thus, global prioritization of EIF2 signaling components, in addition to enrichment within the down regulated transcript cohort of the PC variant, may reflect a cellular strategy of pre-emptive transcriptome recruitment to compensate for catastrophic system stress.

Global gene ontology remodeling precipitated by *calr* deficiency may reflect net effects of discrete contributions made by specific functional CALR regions. To assess this, CALR-truncated variants that exclusively expressed either the protein chaperoning or calcium binding domain were examined for transcriptome changes. Overall, multiple physiological categories were impacted, in line with previous observations reported for diverse gene ontology enrichments in the *calr*^*−/−*^ condition [18]. Broad cellular processes were targeted, with the most enriched disease category for both truncation variants being Cancer. While curation bias is responsible for “Cancer” prioritization in multiple databases [24], functional distribution of CALR outside the endoplasmic reticulum, coupled with roles in cellular adhesion and migration, support the prioritized role of oncological development and progression in these CALR truncation mutants [33, 34].

Targeted focus on cardiac related processes for both CALR truncation variants revealed significant impacts on Cardiac Development and Cardiac Physiology themes, with Cardiovascular Disease uniquely enriched within down regulated genes of the PC variant, which lacks the chaperoning function of full length CALR. Jeopardized expression and/or function of genes within this CALR-truncated transcriptome in a cardiac context identified prioritized molecules that may underlie cardiac pathogenesis precipitated by CALR derangement. The present study identified *Pitx2* as a prioritized gene network hub, i.e. specific promotion of *Pitx2* in Cardiovascular Disease development was observed under conditions of CALR truncation/deficiency. This result suggests a critical role for *Pitx2* within the CALR-regulated cardiogenic network. In the CALR-compromised sub-network, a high betweenness score indicates load and importance of *Pitx2* to the sub-network, while high radiality demonstrates functional relevance and control that *Pitx2* exerts on neighboring nodes [24, 35].

Specific promotion of *Pitx2* in cardiac disease development under conditions of CALR deficiency suggests its critical involvement within the CALR-regulated cardiogenic network. *Pitx2* has been reported to precipitate arrhythmogenesis upon dysregulated expression [36]. Previous studies have identified sinus bradycardia, complete heart block and sudden death associated with dysregulated CALR expression [37, 38]. Here, CALR-dependent remodeling of gene networks that contain *Pitx2*, and gene ontology prioritization of “Cardiac Arrhythmia”, provide bioinformatic evidence that suggest the arrhythmogenic phenotype generated by CALR dysregulation could be mediated by a CALR-PITX2 signaling pathway.

Further data for the critical cardiogenic role of *Pitx2* have been reported in models that express hypomorphic *Pitx2,* which caused disorganization of the atrial septum, increased deposition of extracellular matrix in the atrial chambers, and elongation of the atrioventricular canal [39]. Importantly, *Pitx2* determines right-left heart patterning [40, 41], thus complete loss of *Pitx2* function causes embryonic lethality with associated catastrophic cardiovascular defects [42]. Interestingly, non-cardiac *Pitx2* knockout phenotypes, such as omphalocele, are also present in calreticulin-deficient experimental animal models [42–44]. Together, these observations further support an integrated role for *Pitx2* in CALR-defective phenotypes.

**Fig. 6:**
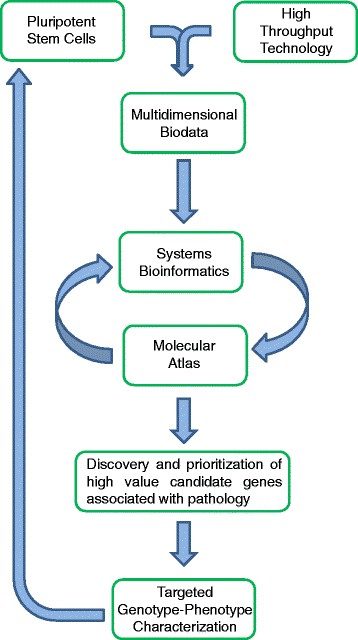
Implementation of recursive bioinformatic strategy for molecular decryption of pathology. The present study employed a bioinformatic heuristic to generate a dynamic molecular atlas used to decipher pathological molecular remodeling. Transcriptome shifts precipitated by truncation variants of a multifunctional protein, CALR, were used here to generate several pluripotent ES cell lines to investigate the putative molecular basis underlying the cardiac phenotype seen in CALR^−/−^ models. By leveraging the large volume processing power of current high throughput technology against the rich transcriptomic landscape of pluripotent ES cells, a deep dataset is produced that can be bioinformatically mined to specified depths to extract features critical for construction of a comprehensive molecular atlas. For example, systems biology methodologies which examine gene expression significance, functional ontology remodeling, and network cartography features can be used to discover and prioritize high value candidate genes for genotype-phenotype characterization. Recursive application, through generation of new pluripotent stem cells derived from targeted candidate genes, ultimately contribute to the molecular atlas that can be used to navigate molecular underpinnings of pathology

Molecular signatures of disease are presently resolved by static indices and molecular panels comprised of experimentally validated biomarkers that diagnose patho-susceptible cells or tissues. This approach is limited by marker selection bias that cannot account for, or provide insight into, global dynamics that sustain disease states. Here, high throughput profiling interrogates dynamic gene expression profiles that offer a depth of resolution unmatched by traditional biomarker panels. Stem cells are natural molecular registries that provide readily abstracted, information-rich transcriptomes that capture molecular underpinnings of discrete cellular phenotypes [18]. Leveraging modern bioinformatic analytics against intricate stem cell transcriptome readouts can extract systems biology features intractable to other approaches, and demonstrates a novel capacity for stem cells as advanced high resolution diagnostic tools (Fig. 6). Molecular cartography of transcript level differences between transcriptomes of pluripotent and phenotype-committed cells can thus prioritize genes critical to disease processes, identifying molecular candidates with potential roles in pathological etiology.

## Conclusions

Pleiotropic responses to single gene lesions recruit a diversity of transcriptome elements that dictate developmental progress, and such dynamic expression changes creates a complex network that make identification of inconspicuous gene relationships challenging. Here, application of a systems biology-derived algorithm of bioinformatic dissection identified discrete gene ontology remodeling effects ascribed to either chaperone or calcium binding regions of CALR. Furthermore, expression changes of a *Pitx2-*inclusive cardiogenic sub-transcriptome were revealed downstream of *calr* deletion, providing novel bioinformatic support for cardiogenic disruption subsequent to *calr* derangement. This paradigm of systems deconvolution thus demonstrates that bioinformatic navigation of an intricate gene expression landscape identifies specific sub-transcriptome remodeling which enhances molecular resolution to expose novel elements of global pathways underlying development and disease processes.

## Methods

### Cell culture, transcriptome isolation and microarray profiling

Independent biological replicates of WT (*n* = 2), *calr*^*−/−*^ (*n* = 5)*,* and *calr* truncation mutants NP (*n* = 3) and PC (*n* = 3) were cultured on 0.1 % gelatin-coated 10 cm dishes in 7.5 % FBS in DMEM supplemented with L-glutamine, NEAA, penicillin/streptomycin, LIF, and BME. Media was changed regularly every 24 or 48 h. Cells reaching ~80 % confluency were passaged 1:10 to maintain proliferative undifferentiated state of all cell lines, as previously performed [18]. Total RNA isolation from embryonic stem cells was performed using the Micro-to-Midi Total RNA Purification System (Invitrogen, Carlsbad, CA) for analysis of transcriptome dynamics. Double stranded complementary cDNA and labeled complementary cRNA were obtained from isolated total RNA, with the latter hybridized against the Mouse 430 2.0 GeneChip (Affymetrix). Arrays were scanned using an argon-ion laser, and visualized using MAS 5.0 Affymetrix software to assess quality of hybridization. Data were deposited to the Gene Expression Omnibus (GEO) repository under accession number GSE13805, with relevant updates.

### Transcript expression analysis

Gene expression data (GEO ID: GSE13805) for calreticulin-deficient embryonic stem cells versus wild type counterparts were analyzed as described previously [9]. To assess the transcript profile changes in calreticulin-truncated mutants, samples were analyzed using Genespring GX to visualize expression dynamics and extract significant differences in each of the truncation mutants compared to wild type control embryonic stem cells. Metaprofile segregation was performed via SOM clustering using Euclidean distance to identify 12 prevalent expression trends among the 4 transcriptomes sampled. Metaprofiles represented by a trendline were plotted in a hexagonal U-Matrix that provides information on each cluster as well as relationship between clusters.

Expression heatmaps with hierarchical dendrograms were used to establish molecular fingerprints for all samples, and were generated using the Pearson coefficient statistic (*r*) applied to determine correlation between gene pairs in each condition as follows:

$$ r=\frac{{\displaystyle \sum_{i=1}^n\left({A}_i-\overline{A}\right)\left({B}_i-\overline{B}\right)}}{\sqrt{\left({\displaystyle \sum_{i=1}^n{\left({A}_i-\overline{A}\right)}^2}\right)}\left({\displaystyle \sum_{i=1}^n{\left({B}_i-\overline{B}\right)}^2}\right)} $$

Equation 1. Summation notation for Pearson coefficient used to establish molecular fingerprint of *calr*^−/−^ stem cells.

In Equation 1, (*A*) and (*B*) are respective sample means for genes *A*_*i*_ and *B*_*i*_ for sample (*i*) out of the total number of samples (*n*), with standard deviation terms for *A*_*i*_ and *B*_*i*_ used as denominator. Condition clustering was performed to determine sample similarity using Euclidean distance as a measure of sample “nearness” [45], and plotted as gene heatmaps. Gene expression changes were profiled using volcano plot analysis, which calculates significance (*p*-value) and fold-change for each quality-filtered transcript, and visualizes these changes in a plot of –log (*p*-value) *versus* log (fold change).

### Functional category extraction and network cartography

To extract the gene expression network framing the transcriptome signature in WT versus *calr* mutant embryonic stem cells, quality filtered transcripts were analyzed using Ingenuity Pathways Analysis to identify prioritized signaling pathways and molecular themes enriched by transcriptome dynamics. Curated molecular interactions among differentially expressed genes were mapped for CALR-variant cell lines versus WT. Global functional categories associated with quality filtered genes were identified in the Ingenuity Pathways Knowledge Base and ranked with a right-tailed Fisher’s exact test. *P*-values for enriched developmental categories were transformed into their –log values and used to generate significance plots, to visualize prioritization in CALR-variant transcriptomes. Cardiac-specific transcriptome meta-analysis was investigated using DAVID (http://david.abcc.ncifcrf.gov/) for independent corroboration of gene prioritizations and correlated Gene Ontology enrichments identified in Ingenuity Pathways Analysis.

Topographical properties of selected networks were examined using the NetworkAnalyzer tool in Cytoscape v3.1.0 to identify degree, degree distribution, betweenness centrality, and radiality. *Degree* – the number of connections (called *edges*) within a network. *Degree distribution* – plot of nodes *versus* degree that identifies which nodes have the greatest number of connections within a network. *Betweenness centrality* – metric that reports which node has the greatest influence on informational flow within a network, i.e. – how connected a node is. *Radiality –* metric which measures the probability of a node being central to the regulation of its network neighbors and indicates functional relevancy.

## Additional files

Additional file 1:
**Functional enrichment data.** *Clustering Data*: Provided are signaling pathways and gene networks enriched in each cluster, as well as gene IDs for all transcripts identified in the UMatrix analysis. *Gene Ontology Data*: Summarization of over represented functional themes in down and up regulated sub-transcriptomes for each of the truncation variants. Additional file 2: Table S1. Functional enrichment of physiological categories within up and down regulated transcriptomes of the NP truncation variant. Provided are p-values for each category, with systems uniquely regulated shown below. Additional file 3: Figure S1. Physiological systems impacted by CALR^-/-^-PC. Extraction of physiological development categories enriched in the CALR^-/-^-PC transcriptome revealed prioritization of Cardiovascular System Development and Function, independent of up or down regulated gene expression trends. Enrichment *P*-values for each physiological category in the up and down regulated sub-transcriptome cohorts were transformed into their –log values and plotted for ease of visualization. Additional file 4: Table S2. Functional enrichment of physiological categories within up and down regulated transcriptomes of the PC truncation variant. Provided are p-values for each category, with systems uniquely regulated in either the up or down regulated transcriptome cohort listed below. Additional file 5: Table S3. Genes that enriched for Cardiovascular Disease within the down regulated cohort of transcripts isolated from the PC variant. Provided are Affymetrix Probeset IDs and gene names.
