# Supplementary material for: Systems biology surveillance decrypts pathological transcriptome remodeling
Source: BMC Syst Biol. 2015 Jul 17;9:36. doi: 10.1186/s12918-015-0177-8 (PMC4504166; doi:10.1186/s12918-015-0177-8)
Supplement: Additional file 1: — Functional enrichment data. Clustering Data: Provided are signaling pathways and gene networks enriched in each cluster, as well as gene IDs for all transcripts identified in the UMatrix analysis. Gene Ontology Data: Summarization of over represented functional themes in down and up regulated sub-transcriptomes for each of the truncation variants. [file 12918_2015_177_MOESM1_ESM.zip › 9929599221407335_add1.pdf]

Analysis Name: Cluster 1 - 2014-06-04 08:01 PM
Analysis Creation Date: 2014-06-04
Build version: 308606M
Content version: 18488943 (Release Date: 2014-03-23)

Analysis settings

View

Reference set: Mouse Genome 430 2.0 Array

Relationship to include: Direct and Indirect

Includes Endogenous Chemicals

Optional Analyses: My Pathways My List

Filter Summary:

Consider only relationships where

confidence = Experimentally Observed

Cutoff:

(c) 2000-2014 Ingenuity Systems, Inc. All rights reserved.

Top Canonical Pathways

Name	p-value	Ratio
GDP-mannose Biosynthesis	3.29E-03	2/13 (0.154)
NGF Signaling	4.77E-03	6/122 (0.049)
Ephrin A Signaling	5.49E-03	4/54 (0.074)
ATM Signaling	1.15E-02	4/66 (0.061)
Cleavage and Polyadenylation of Pre-mRNA	1.36E-02	2/13 (0.154)

Top Upstream Regulators

Upstream Regulator	p-value of overlap	Predicted Activation State
CDK4	1.37E-07	
HOXA9	1.00E-04	
CCND1	1.49E-04	
miR-17-5p (and other miRNAs w/seed AAAGUGC)	1.12E-03	
GNA12	1.54E-03	

(c) 2000-2014 Ingenuity Systems, Inc. All rights reserved.

INGENUITY® SYSTEMS

Top Diseases and Bio Functions

Diseases and Disorders

Name	p-value	# Molecules
Cancer	1.20E-12 - 1.51E-02	191
Gastrointestinal Disease	2.48E-11 - 1.51E-02	108
Organismal Injury and Abnormalities	5.13E-06 - 1.51E-02	100
Reproductive System Disease	5.13E-06 - 7.67E-03	88
Respiratory Disease	4.59E-05 - 1.51E-02	33

Molecular and Cellular Functions

Name	p-value	# Molecules
Post-Translational Modification	4.99E-06 - 7.67E-03	41
Cellular Development	6.21E-06 - 1.51E-02	72
Cellular Growth and Proliferation	6.21E-06 - 1.51E-02	89
Cell Cycle	1.48E-05 - 1.51E-02	55
Cellular Assembly and Organization	1.48E-05 - 1.51E-02	66

Physiological System Development and Function

Name	p-value	# Molecules
Skeletal and Muscular System Development and Function	1.47E-04 - 1.27E-02	21
Connective Tissue Development and Function	2.28E-04 - 1.51E-02	15
Nervous System Development and Function	2.28E-04 - 1.51E-02	36
Tissue Development	2.28E-04 - 1.51E-02	56
Tissue Morphology	2.28E-04 - 1.51E-02	26

(c) 2000-2014 Ingenuity Systems, Inc. All rights reserved.

INGENUITY® SYSTEMS

Top Tox Functions

Assays: Clinical Chemistry and Hematology

Name	p-value	# Molecules
Increased Levels of Bilirubin	3.01E-02 - 3.01E-02	1
Increased Levels of ALT	7.35E-02 - 7.35E-02	1
Increased Levels of AST	8.75E-02 - 8.75E-02	1
Increased Levels of Red Blood Cells	1.56E-01 - 1.56E-01	3
Increased Levels of Hematocrit	3.91E-01 - 3.91E-01	2

Cardiotoxicity

Name	p-value	# Molecules
Cardiac Arrythmia	1.51E-02 - 4.69E-01	5
Pulmonary Hypertension	1.51E-02 - 1.08E-01	2
Cardiac Hypertrophy	4.32E-02 - 1.00E00	5
Cardiac Necrosis/Cell Death	6.26E-02 - 2.98E-01	7
Cardiac Enlargement	7.35E-02 - 8.75E-02	2

Hepatotoxicity

Name	p-value	# Molecules
Hepatocellular Carcinoma	5.11E-04 - 2.74E-01	12
Liver Hyperplasia/Hyperproliferation	5.11E-04 - 2.74E-01	17
Liver Proliferation	2.36E-03 - 2.09E-01	7
Liver Enlargement	3.01E-02 - 7.35E-02	2
Liver Necrosis/Cell Death	3.01E-02 - 1.93E-01	6

Nephrotoxicity

(c) 2000-2014 Ingenuity Systems, Inc. All rights reserved.

INGENUITY SYSTEMS

Name	p-value	# Molecules
Renal Destruction	1.51E-02 - 1.51E-02	1
Renal Lysis	1.51E-02 - 1.51E-02	1
Renal Hypertrophy	1.60E-02 - 1.60E-02	2
Renal Regeneration	3.01E-02 - 3.01E-02	1
Glomerular Injury	5.92E-02 - 5.92E-02	1

Top Regulator Effect Networks

Top Networks

ID	Associated Network Functions	Score
1	RNA Post-Transcriptional Modification, Cellular Assembly and Organization, Cellular Compromise	66
2	Cellular Assembly and Organization, Cellular Function and Maintenance, Cancer	38
3	Cell Morphology, Cellular Assembly and Organization, Cellular Function and Maintenance	36
4	Gene Expression, Post-Translational Modification, Cell Cycle	34
5	Cancer, Gastrointestinal Disease, Neurological Disease	29

Top Tox Lists

Name	p-value	Ratio
Liver Proliferation	3.84E-02	7/216 (0.032)
Increases Cardiac Dilation	5.45E-02	2/28 (0.071)
Cardiac Necrosis/Cell Death	6.69E-02	7/253 (0.028)
Anti-Apoptosis	7.53E-02	2/32 (0.062)
NF-κB Signaling	1.14E-01	5/211 (0.024)

(c) 2000-2014 Ingenuity Systems, Inc. All rights reserved.

INGENUITY® SYSTEMS

Top My Lists

Name	p-value	Ratio

Top My Pathways

Name	p-value	Ratio

Top Molecules

This analysis has no expression values.

(c) 2000-2014 Ingenuity Systems, Inc. All rights reserved.
