# Supplementary material for: Systems biology surveillance decrypts pathological transcriptome remodeling
Source: BMC Syst Biol. 2015 Jul 17;9:36. doi: 10.1186/s12918-015-0177-8 (PMC4504166; doi:10.1186/s12918-015-0177-8)
Supplement: Additional file 1: — Functional enrichment data. Clustering Data: Provided are signaling pathways and gene networks enriched in each cluster, as well as gene IDs for all transcripts identified in the UMatrix analysis. Gene Ontology Data: Summarization of over represented functional themes in down and up regulated sub-transcriptomes for each of the truncation variants. [file 12918_2015_177_MOESM1_ESM.zip › 9929599221407335_add10.pdf]

Interactive pathway analysis of complex 'omics data

Analysis Name: Cluster 10 - 2014-06-04 08:15 PM
Analysis Creation Date: 2014-06-04
Build version: 308606M
Content version: 18488943 (Release Date: 2014-03-23)

Analysis settings

View

Reference set: Mouse Genome 430 2.0 Array

Relationship to include: Direct and Indirect

Includes Endogenous Chemicals

Optional Analyses: My Pathways My List

Filter Summary:

Consider only relationships where

confidence = Experimentally Observed

Cutoff:

(c) 2000-2014 Ingenuity Systems, Inc. All rights reserved.

Top Canonical Pathways

Name	p-value	Ratio
Oxidative Phosphorylation	4.52E-04	10/120 (0.083)
Calcium-induced T Lymphocyte Apoptosis	1.3E-03	7/71 (0.099)
Nur77 Signaling in T Lymphocytes	2.47E-03	6/64 (0.094)
Role of NFAT in Regulation of the Immune Response	3.33E-03	12/200 (0.06)
Type I Diabetes Mellitus Signaling	3.52E-03	9/121 (0.074)

Top Upstream Regulators

Upstream Regulator	p-value of overlap	Predicted Activation State
POR	1.03E-03	
exisulind	5.74E-03	
asparagine	5.74E-03	
RNF6	5.74E-03	
PFKFB3	5.74E-03	

(c) 2000-2014 Ingenuity Systems, Inc. All rights reserved.

INGENUITY® SYSTEMS

Top Diseases and Bio Functions

Diseases and Disorders

Name	p-value	# Molecules
Cancer	2.80E-03 - 4.36E-02	45
Dermatological Diseases and Conditions	2.99E-03 - 3.09E-02	11
Gastrointestinal Disease	2.99E-03 - 3.64E-02	16
Immunological Disease	2.99E-03 - 3.09E-02	20
Inflammatory Disease	2.99E-03 - 3.64E-02	11

Molecular and Cellular Functions

Name	p-value	# Molecules
Molecular Transport	2.80E-04 - 3.09E-02	41
Cell Death and Survival	6.39E-04 - 4.31E-02	41
RNA Post-Transcriptional Modification	1.95E-03 - 1.95E-03	4
Cellular Compromise	2.80E-03 - 3.64E-02	15
Cellular Growth and Proliferation	2.80E-03 - 4.36E-02	27

Physiological System Development and Function

Name	p-value	# Molecules
Tumor Morphology	2.80E-03 - 3.09E-02	18
Nervous System Development and Function	4.02E-03 - 3.64E-02	20
Tissue Development	4.02E-03 - 3.67E-02	30
Auditory and Vestibular System Development and Function	4.68E-03 - 3.09E-02	7
Renal and Urological System Development and Function	5.24E-03 - 3.09E-02	6

(c) 2000-2014 Ingenuity Systems, Inc. All rights reserved.

INGENUITY® SYSTEMS

Top Tox Functions

Assays: Clinical Chemistry and Hematology

Name	p-value	# Molecules
Decreased Levels of Potassium	5.49E-03 - 5.49E-03	2
Increased Levels of Albumin	3.09E-02 - 1.72E-01	2
Increased Levels of Bilirubin	6.09E-02 - 6.09E-02	1
Increased Levels of Potassium	6.09E-02 - 2.22E-01	2
Decreased Levels of Albumin	1.72E-01 - 1.72E-01	1

Cardiotoxicity

Name	p-value	# Molecules
Cardiac Necrosis/Cell Death	2.99E-02 - 5.72E-01	12
Cardiac Proliferation	3.09E-02 - 3.60E-01	5
Cardiac Enlargement	3.64E-02 - 1.72E-01	3
Cardiac Hyperplasia/Hyperproliferation	3.64E-02 - 3.64E-02	2
Cardiac Degeneration	6.09E-02 - 2.22E-01	2

Hepatotoxicity

Name	p-value	# Molecules
Hepatocellular Carcinoma	3.09E-02 - 5.37E-01	12
Liver Hyperplasia/Hyperproliferation	3.09E-02 - 1.00E00	18
Hepatocellular Peroxisome Proliferation	3.64E-02 - 1.18E-01	2
Liver Hyperbilirubinemia	6.09E-02 - 6.09E-02	1
Liver Degeneration	6.43E-02 - 6.43E-02	3

Nephrotoxicity

(c) 2000-2014 Ingenuity Systems, Inc. All rights reserved.

INGENUITY SYSTEMS

Name	p-value	# Molecules
Glomerular Injury	1.69E-02 - 1.18E-01	7
Nephrosis	3.09E-02 - 2.58E-01	3
Renal Proliferation	3.09E-02 - 5.58E-01	4
Renal Degradation	6.09E-02 - 6.09E-02	1
Renal Inflammation	6.09E-02 - 1.00E00	6

Top Regulator Effect Networks

Top Networks

ID	Associated Network Functions	Score
1	Organismal Development, Developmental Disorder, Hereditary Disorder	60
2	Hereditary Disorder, Neurological Disease, Cancer	49
3	Cell Cycle, Lipid Metabolism, Small Molecule Biochemistry	40
4	Carbohydrate Metabolism, Small Molecule Biochemistry, Nucleic Acid Metabolism	37
5	Hereditary Disorder, Metabolic Disease, Neurological Disease	33

Top Tox Lists

Name	p-value	Ratio
Mitochondrial Dysfunction	5.67E-03	11/169 (0.065)
Cholesterol Biosynthesis	1.01E-02	3/16 (0.188)
NF-κB Signaling	3.72E-02	10/211 (0.047)
Cardiac Necrosis/Cell Death	6.16E-02	12/253 (0.047)
NRF2-mediated Oxidative Stress Response	1.12E-01	10/234 (0.043)

(c) 2000-2014 Ingenuity Systems, Inc. All rights reserved.

INGENUITY® SYSTEMS

Top My Lists

Name	p-value	Ratio

Top My Pathways

Name	p-value	Ratio

Top Molecules

This analysis has no expression values.

(c) 2000-2014 Ingenuity Systems, Inc. All rights reserved.

INGENUITY® SYSTEMS
