# Supplementary material for: Systems biology surveillance decrypts pathological transcriptome remodeling
Source: BMC Syst Biol. 2015 Jul 17;9:36. doi: 10.1186/s12918-015-0177-8 (PMC4504166; doi:10.1186/s12918-015-0177-8)
Supplement: Additional file 1: — Functional enrichment data. Clustering Data: Provided are signaling pathways and gene networks enriched in each cluster, as well as gene IDs for all transcripts identified in the UMatrix analysis. Gene Ontology Data: Summarization of over represented functional themes in down and up regulated sub-transcriptomes for each of the truncation variants. [file 12918_2015_177_MOESM1_ESM.zip › 9929599221407335_add11.pdf]

Analysis Name: Cluster 11 - 2014-06-04 08:15 PM
Analysis Creation Date: 2014-06-04
Build version: 308606M
Content version: 18488943 (Release Date: 2014-03-23)

Analysis settings

View

Reference set: Mouse Genome 430 2.0 Array

Relationship to include: Direct and Indirect

Includes Endogenous Chemicals

Optional Analyses: My Pathways My List

Filter Summary:

Consider only relationships where

confidence = Experimentally Observed

Cutoff:

(c) 2000-2014 Ingenuity Systems, Inc. All rights reserved.

Top Canonical Pathways

Name	p-value	Ratio
EIF2 Signaling	3.41E-07	16/201 (0.08)
Regulation of eIF4 and p70S6K Signaling	2.32E-03	9/175 (0.051)
Gαs Signaling	7.26E-03	7/125 (0.056)
Eicosanoid Signaling	9.48E-03	5/86 (0.058)
Colorectal Cancer Metastasis Signaling	1.09E-02	11/268 (0.041)

Top Upstream Regulators

Upstream Regulator	p-value of overlap	Predicted Activation State
RORC	5.08E-05	
PTTG1IP	1.33E-03	
FCGR2B	1.81E-03	
COMMD3-BMI1	2.06E-03	
LGR4	3.57E-03	

(c) 2000-2014 Ingenuity Systems, Inc. All rights reserved.

Top Diseases and Bio Functions

Diseases and Disorders

Name	p-value	# Molecules
Cancer	1.43E-03 - 3.21E-02	76
Developmental Disorder	2.00E-03 - 2.75E-02	34
Endocrine System Disorders	2.00E-03 - 3.26E-02	10
Metabolic Disease	2.00E-03 - 2.75E-02	14
Inflammatory Response	4.62E-03 - 3.54E-02	15

Molecular and Cellular Functions

Name	p-value	# Molecules
Cell Death and Survival	1.02E-04 - 3.27E-02	20
Cell Cycle	4.83E-04 - 3.54E-02	15
Cellular Development	5.51E-04 - 3.45E-02	35
Cellular Growth and Proliferation	5.51E-04 - 3.01E-02	26
Cell-To-Cell Signaling and Interaction	8.43E-04 - 3.21E-02	20

Physiological System Development and Function

Name	p-value	# Molecules
Embryonic Development	2.01E-04 - 2.90E-02	30
Organismal Development	2.01E-04 - 2.90E-02	29
Tissue Development	2.01E-04 - 3.21E-02	36
Hematological System Development and Function	5.51E-04 - 3.45E-02	29
Connective Tissue Development and Function	8.43E-04 - 3.54E-02	16

(c) 2000-2014 Ingenuity Systems, Inc. All rights reserved.

INGENUITY® SYSTEMS

Top Tox Functions

Assays: Clinical Chemistry and Hematology

Name	p-value	# Molecules
Increased Levels of Blood Urea Nitrogen	3.69E-02 - 3.69E-02	2
Increased Levels of Bilirubin	4.35E-02 - 4.35E-02	1
Increased Levels of Creatinine	4.73E-02 - 2.68E-01	4
Decreased Levels of Albumin	8.51E-02 - 8.51E-02	1
Increased Levels of Potassium	1.63E-01 - 3.00E-01	2

Cardiotoxicity

Name	p-value	# Molecules
Cardiac Arrythmia	2.20E-02 - 1.00E00	3
Cardiac Inflammation	2.20E-02 - 5.99E-01	2
Congenital Heart Anomaly	2.20E-02 - 5.10E-01	5
Cardiac Proliferation	4.35E-02 - 5.55E-01	2
Cardiac Transformation	4.35E-02 - 4.35E-02	1

Hepatotoxicity

Name	p-value	# Molecules
Liver Degradation	2.20E-02 - 2.20E-02	1
Liver Inflammation/Hepatitis	2.20E-02 - 4.76E-01	7
Glutathione Depletion In Liver	4.19E-02 - 2.68E-01	3
Liver Cirrhosis	6.46E-02 - 5.86E-01	5
Liver Hypertrophy	6.46E-02 - 6.46E-02	1

Nephrotoxicity

(c) 2000-2014 Ingenuity Systems, Inc. All rights reserved.

INGENUITY® SYSTEMS

Name	p-value	# Molecules
Renal Degeneration	2.20E-02 - 2.20E-02	1
Renal Depletion	2.20E-02 - 2.20E-02	1
Renal Inflammation	2.20E-02 - 4.27E-01	7
Renal Nephritis	2.20E-02 - 4.27E-01	7
Nephrosis	4.35E-02 - 3.58E-01	2

Top Regulator Effect Networks

Top Networks

ID	Associated Network Functions	Score
1	Cancer, Hematological Disease, Embryonic Development	51
2	Cellular Development, Hematological System Development and Function, Hematopoiesis	37
3	Cell Morphology, Nervous System Development and Function, Cellular Assembly and Organization	30
4	Antigen Presentation, Inflammatory Response, Cellular Movement	24
5	Hematological Disease, Cell-To-Cell Signaling and Interaction, Cell Signaling	23

Top Tox Lists

Name	p-value	Ratio
Cytochrome P450 Panel - Substrate is a Fatty Acid (Human)	1.57E-02	2/10 (0.2)
Xenobiotic Metabolism Signaling	2.31E-02	12/336 (0.036)
Cytochrome P450 Panel - Substrate is a Fatty Acid (Rat)	2.33E-02	2/11 (0.182)
Hepatic Cholestasis	2.77E-02	7/142 (0.049)
Cytochrome P450 Panel - Substrate is a Fatty Acid (Mouse)	3.69E-02	2/14 (0.143)

(c) 2000-2014 Ingenuity Systems, Inc. All rights reserved.

INGENUITY SYSTEMS

Top My Lists

Name	p-value	Ratio

Top My Pathways

Name	p-value	Ratio

Top Molecules

This analysis has no expression values.

(c) 2000-2014 Ingenuity Systems, Inc. All rights reserved.

INGENUITY® SYSTEMS
