# Supplementary material for: Systems biology surveillance decrypts pathological transcriptome remodeling
Source: BMC Syst Biol. 2015 Jul 17;9:36. doi: 10.1186/s12918-015-0177-8 (PMC4504166; doi:10.1186/s12918-015-0177-8)
Supplement: Additional file 1: — Functional enrichment data. Clustering Data: Provided are signaling pathways and gene networks enriched in each cluster, as well as gene IDs for all transcripts identified in the UMatrix analysis. Gene Ontology Data: Summarization of over represented functional themes in down and up regulated sub-transcriptomes for each of the truncation variants. [file 12918_2015_177_MOESM1_ESM.zip › 9929599221407335_add12.pdf]

Interactive pathway analysis of complex 'omics data

Analysis Name: Cluster 12 - 2014-06-04 08:16 PM
Analysis Creation Date: 2014-06-04
Build version: 308606M
Content version: 18488943 (Release Date: 2014-03-23)

Analysis settings

View

Reference set: Mouse Genome 430 2.0 Array

Relationship to include: Direct and Indirect

Includes Endogenous Chemicals

Optional Analyses: My Pathways My List

Filter Summary:

Consider only relationships where

confidence = Experimentally Observed

Cutoff:

(c) 2000-2014 Ingenuity Systems, Inc. All rights reserved.

Top Canonical Pathways

Name	p-value	Ratio
Glioma Signaling	6.1E-04	6/113 (0.053)
EIF2 Signaling	1.98E-03	7/201 (0.035)
Chronic Myeloid Leukemia Signaling	3.7E-03	5/106 (0.047)
Role of BRCA1 in DNA Damage Response	4.7E-03	4/71 (0.056)
Cell Cycle: G1/S Checkpoint Regulation	4.7E-03	4/72 (0.056)

Top Upstream Regulators

Upstream Regulator	p-value of overlap	Predicted Activation State
U0126	1.90E-06	
TGFB1	1.95E-06	
beta-estradiol	3.31E-06	
MYCN	3.92E-06	
FGF2	4.85E-06	

(c) 2000-2014 Ingenuity Systems, Inc. All rights reserved.

INGENUITY® SYSTEMS

Top Diseases and Bio Functions

Diseases and Disorders

Name	p-value	# Molecules
Cancer	7.82E-08 - 1.13E-02	90
Organismal Injury and Abnormalities	2.77E-05 - 1.13E-02	62
Developmental Disorder	2.77E-04 - 1.13E-02	17
Ophthalmic Disease	2.77E-04 - 1.13E-02	10
Reproductive System Disease	3.92E-04 - 1.13E-02	44

Molecular and Cellular Functions

Name	p-value	# Molecules
Cellular Growth and Proliferation	3.64E-07 - 1.13E-02	72
Cellular Movement	1.11E-06 - 1.09E-02	48
Cellular Development	3.08E-05 - 1.13E-02	56
Cell-To-Cell Signaling and Interaction	3.88E-05 - 1.13E-02	31
Cell Morphology	7.61E-05 - 1.13E-02	44

Physiological System Development and Function

Name	p-value	# Molecules
Cardiovascular System Development and Function	1.68E-05 - 1.13E-02	46
Connective Tissue Development and Function	3.08E-05 - 1.13E-02	32
Tissue Development	3.08E-05 - 1.13E-02	58
Nervous System Development and Function	7.61E-05 - 1.13E-02	32
Organismal Development	1.10E-04 - 1.13E-02	66

(c) 2000-2014 Ingenuity Systems, Inc. All rights reserved.

INGENUITY SYSTEMS

Top Tox Functions

Assays: Clinical Chemistry and Hematology

Name	p-value	# Molecules
Increased Levels of Red Blood Cells	3.53E-03 - 3.53E-03	5
Increased Levels of Albumin	2.25E-02 - 2.25E-02	1
Decreased Levels of Albumin	6.60E-02 - 6.60E-02	1
Increased Levels of Hematocrit	7.99E-02 - 7.99E-02	3
Increased Levels of LDH	9.73E-02 - 9.73E-02	1

Cardiotoxicity

Name	p-value	# Molecules
Cardiac Arrythmia	1.13E-02 - 3.82E-01	3
Tachycardia	1.13E-02 - 1.13E-02	1
Congenital Heart Anomaly	1.69E-02 - 3.05E-01	5
Heart Failure	2.25E-02 - 5.21E-01	3
Cardiac Fibrosis	3.35E-02 - 3.35E-02	1

Hepatotoxicity

Name	p-value	# Molecules
Hepatocellular Carcinoma	1.13E-02 - 3.94E-01	9
Liver Damage	1.13E-02 - 3.90E-01	6
Liver Hyperplasia/Hyperproliferation	1.13E-02 - 3.94E-01	13
Glutathione Depletion In Liver	1.21E-02 - 1.21E-02	2
Liver Inflammation/Hepatitis	2.09E-02 - 2.81E-01	5

Nephrotoxicity

(c) 2000-2014 Ingenuity Systems, Inc. All rights reserved.

Name	p-value	# Molecules
Renal Proliferation	1.12E-02 - 4.95E-01	7
Renal Damage	1.13E-02 - 3.66E-01	4
Renal Inflammation	2.25E-02 - 5.49E-01	3
Renal Nephritis	2.25E-02 - 5.49E-01	3
Renal Necrosis/Cell Death	3.21E-02 - 2.56E-01	9

Top Regulator Effect Networks

Top Networks

ID	Associated Network Functions	Score
1	Cancer, Cardiovascular System Development and Function, Cellular Development	44
2	Cellular Growth and Proliferation, Cancer, Cellular Development	41
3	Cardiovascular System Development and Function, Cell-To-Cell Signaling and Interaction, Tissue Development	31
4	Developmental Disorder, Hereditary Disorder, Organismal Injury and Abnormalities	28
5	Small Molecule Biochemistry, Hereditary Disorder, Metabolic Disease	27

Top Tox Lists

Name	p-value	Ratio
NRF2-mediated Oxidative Stress Response	6.02E-04	9/234 (0.038)
Cell Cycle: G1/S Checkpoint Regulation	5.29E-03	4/69 (0.058)
Fatty Acid Metabolism	6.26E-03	5/118 (0.042)
Cytochrome P450 Panel - Substrate is a Xenobiotic (Human)	9.14E-03	2/20 (0.1)
Increases Renal Proliferation	1.25E-02	5/128 (0.039)

(c) 2000-2014 Ingenuity Systems, Inc. All rights reserved.

INGENUITY® SYSTEMS

Top My Lists

Name	p-value	Ratio

Top My Pathways

Name	p-value	Ratio

Top Molecules

This analysis has no expression values.

(c) 2000-2014 Ingenuity Systems, Inc. All rights reserved.

INGENUITY® SYSTEMS
