# Supplementary material for: Systems biology surveillance decrypts pathological transcriptome remodeling
Source: BMC Syst Biol. 2015 Jul 17;9:36. doi: 10.1186/s12918-015-0177-8 (PMC4504166; doi:10.1186/s12918-015-0177-8)
Supplement: Additional file 1: — Functional enrichment data. Clustering Data: Provided are signaling pathways and gene networks enriched in each cluster, as well as gene IDs for all transcripts identified in the UMatrix analysis. Gene Ontology Data: Summarization of over represented functional themes in down and up regulated sub-transcriptomes for each of the truncation variants. [file 12918_2015_177_MOESM1_ESM.zip › 9929599221407335_add18.pdf]

IPA® Interactive pathway analysis of complex 'omics data

INGENUITY® SYSTEMS

Analysis Name: DOWN - FC (abs) ([NP] vs [Con- 2013-02-28 01:20 PM
Analysis Creation Date: 2013-02-28
Build version: 302937
Content version: Not available.

Note: Since this analysis was run, curated information for 4% of the molecules has changed.
For the latest information, please run a new analysis on this dataset.

Analysis settings

View

Reference set: Ingenuity Knowledge Base (Genes Only)

Relationship to include: Direct and Indirect

Includes Endogenous Chemicals

Optional Analyses: My Pathways My List

Filter Summary:

Consider only relationships where

data sources = An Open Access Database of Genome-wide Association Results OR BIND OR BIOGRID OR Breast Cancer Information Core (BIC) OR Catalogue Of Somatic Mutations In Cancer (COSMIC) OR Chemical Carcinogenesis Research Information System (CCRIS) OR ClinicalTrials.gov OR ClinVar OR Cognia OR DIP OR DrugBank OR Gene Ontology (GO) OR GVK Biosciences OR Hazardous Substances Data Bank (HSDB) OR HumanCyc OR Ingenuity Expert Findings OR Ingenuity ExpertAssist Findings OR INTACT OR Interactome studies OR MINT OR MIPS OR miRBase OR miRecords OR Mouse Genome Database (MGD) OR Obesity Gene Map Database OR Online Mendelian Inheritance in Man (OMIM) OR TarBase OR TargetScan Human

Cutoff:

(c) 2000-2014 Ingenuity Systems, Inc. All rights reserved.

Top Canonical Pathways

Name	p-value	Ratio
Molecular Mechanisms of Cancer	8.94E-03	22/378 (0.058)
Glioma Signaling	9.34E-03	9/112 (0.08)
ILK Signaling	1.01E-02	14/192 (0.073)
HGF Signaling	1.21E-02	9/105 (0.086)
Glycerol Degradation I	1.31E-02	2/12 (0.167)

Top Upstream Regulators

Upstream Regulator	p-value of overlap	Predicted Activation State
TGFB1	6.27E-08	
miR-4434 (and other miRNAs w/seed GGAGAAG)	4.84E-07	
estrogen receptor	1.25E-06	
HRAS	1.53E-06	
TBL1X	2.93E-06	

(c) 2000-2014 Ingenuity Systems, Inc. All rights reserved.

Top Diseases and Bio Functions

Diseases and Disorders

Name	p-value	# Molecules
Cancer	8.16E-06 - 8.40E-03	206
Organismal Injury and Abnormalities	2.78E-05 - 6.94E-03	50
Inflammatory Response	1.41E-04 - 8.03E-03	30
Developmental Disorder	1.51E-04 - 8.03E-03	87
Gastrointestinal Disease	3.46E-04 - 7.08E-03	30

Molecular and Cellular Functions

Name	p-value	# Molecules
Cellular Assembly and Organization	7.10E-08 - 8.03E-03	127
Cellular Function and Maintenance	7.10E-08 - 8.03E-03	122
Cell Death and Survival	7.15E-08 - 8.03E-03	204
Cellular Growth and Proliferation	2.19E-07 - 6.31E-03	216
Gene Expression	6.07E-07 - 8.03E-03	154

Physiological System Development and Function

Name	p-value	# Molecules
Tissue Morphology	5.69E-07 - 8.03E-03	168
Organismal Survival	7.64E-07 - 7.64E-07	113
Cardiovascular System Development and Function	5.18E-06 - 8.03E-03	106
Embryonic Development	5.92E-06 - 8.40E-03	143
Nervous System Development and Function	5.92E-06 - 8.45E-03	137

(c) 2000-2014 Ingenuity Systems, Inc. All rights reserved.

INGENUITY® SYSTEMS

Top Tox Functions

Assays: Clinical Chemistry and Hematology

Name	p-value	# Molecules
Increased Levels of Albumin	7.37E-02 - 7.37E-02	1
Decreased Levels of Albumin	1.42E-01 - 2.05E-01	2
Increased Levels of Red Blood Cells	1.47E-01 - 1.47E-01	6
Increased Levels of Hematocrit	1.58E-01 - 1.58E-01	6
Increased Levels of LDH	2.35E-01 - 2.35E-01	1

Cardiotoxicity

Name	p-value	# Molecules
Cardiac Arrythmia	4.13E-03 - 5.53E-01	10
Cardiac Proliferation	1.58E-02 - 2.64E-01	7
Tachycardia	3.71E-02 - 4.79E-01	6
Cardiac Inflammation	3.76E-02 - 4.79E-01	5
Congenital Heart Anomaly	3.76E-02 - 4.58E-01	7

Hepatotoxicity

Name	p-value	# Molecules
Liver Enlargement	1.41E-03 - 1.41E-03	2
Liver Necrosis/Cell Death	1.41E-03 - 3.53E-01	11
Liver Dysplasia	1.91E-02 - 7.37E-02	2
Liver Proliferation	2.82E-02 - 4.15E-01	9
Liver Cholestasis	3.76E-02 - 5.02E-01	5

(c) 2000-2014 Ingenuity Systems, Inc. All rights reserved.

Nephrotoxicity

Name	p-value	# Molecules
Renal Necrosis/Cell Death	6.30E-05 - 1.00E00	27
Renal Proliferation	6.31E-03 - 1.74E-01	14
Nephrosis	3.76E-02 - 5.43E-01	2
Renal Inflammation	3.76E-02 - 1.00E00	6
Renal Nephritis	3.76E-02 - 1.00E00	6

Top Regulator Effect Networks

Top Networks

ID	Associated Network Functions	Score
1	Hematological Disease, Organismal Injury and Abnormalities, Cellular Compromise	49
2	Cellular Compromise, DNA Replication, Recombination, and Repair, Cell Death and Survival	42
3	Cellular Assembly and Organization, Cellular Function and Maintenance, Endocrine System Development and Function	33
4	Organismal Development, DNA Replication, Recombination, and Repair, Reproductive System Development and Function	30
5	Cellular Movement, Immune Cell Trafficking, Cellular Growth and Proliferation	28

Top Tox Lists

Name	p-value	Ratio
Renal Necrosis/Cell Death	7.96E-03	27/437 (0.062)
Hypoxia-Inducible Factor Signaling	1.58E-02	7/70 (0.1)

(c) 2000-2014 Ingenuity Systems, Inc. All rights reserved.

Increases Renal Proliferation	1.93E-02	9/107 (0.084)
Hepatic Cholestasis	1.99E-02	11/144 (0.076)
Acute Renal Failure Panel (Rat)	2.84E-02	6/62 (0.097)

(c) 2000-2014 Ingenuity Systems, Inc. All rights reserved.

INGENUITY® SYSTEMS

Top My Lists

Name	p-value	Ratio

Top My Pathways

Name	p-value	Ratio

Top Molecules

This analysis has no expression values.

(c) 2000-2014 Ingenuity Systems, Inc. All rights reserved.

INGENUITY®
SYSTEMS
