# Supplementary material for: Systems biology surveillance decrypts pathological transcriptome remodeling
Source: BMC Syst Biol. 2015 Jul 17;9:36. doi: 10.1186/s12918-015-0177-8 (PMC4504166; doi:10.1186/s12918-015-0177-8)
Supplement: Additional file 1: — Functional enrichment data. Clustering Data: Provided are signaling pathways and gene networks enriched in each cluster, as well as gene IDs for all transcripts identified in the UMatrix analysis. Gene Ontology Data: Summarization of over represented functional themes in down and up regulated sub-transcriptomes for each of the truncation variants. [file 12918_2015_177_MOESM1_ESM.zip › 9929599221407335_add19.pdf]

Analysis Name: DOWN - FC (abs) ([PC] vs [Con- 2013-02-28 01:22 PM
Analysis Creation Date: 2013-02-28
Build version: 302937
Content version: Not available.

Note: Since this analysis was run, curated information for 4% of the molecules has changed.
For the latest information, please run a new analysis on this dataset.

Analysis settings

View
Reference set: Ingenuity Knowledge Base (Genes Only)
Relationship to include: Direct and Indirect
Includes Endogenous Chemicals
Optional Analyses: My Pathways My List

Filter Summary:
Consider only relationships where
data sources = An Open Access Database of Genome-wide Association Results OR BIND OR BIOGRID OR Breast Cancer Information Core (BIC) OR Catalogue Of Somatic Mutations In Cancer (COSMIC) OR Chemical Carcinogenesis Research Information System (CCRIS) OR ClinicalTrials.gov OR ClinVar OR Cognia OR DIP OR DrugBank OR Gene Ontology (GO) OR GVK Biosciences OR Hazardous Substances Data Bank (HSDB) OR HumanCyc OR Ingenuity Expert Findings OR Ingenuity ExpertAssist Findings OR INTACT OR Interactome studies OR MINT OR MIPS OR miRBase OR miRecords OR Mouse Genome Database (MGD) OR Obesity Gene Map Database OR Online Mendelian Inheritance in Man (OMIM) OR TarBase OR TargetScan Human
Cutoff:

(c) 2000-2014 Ingenuity Systems, Inc. All rights reserved.

Top Canonical Pathways

Name	p-value	Ratio
EIF2 Signaling	1.18E-03	17/200 (0.085)
Glioma Signaling	1.44E-03	11/112 (0.098)
Glioblastoma Multiforme Signaling	2.2E-03	14/164 (0.085)
Molecular Mechanisms of Cancer	2.45E-03	25/378 (0.066)
Epithelial Adherens Junction Signaling	5.54E-03	13/147 (0.088)

Top Upstream Regulators

Upstream Regulator	p-value of overlap	Predicted Activation State
estrogen receptor	3.32E-07	
TGFB1	7.30E-07	
miR-4271 (and other miRNAs w/seed GGGGAAG)	9.22E-07	
forskolin	1.74E-06	
miR-4434 (and other miRNAs w/seed GGAGAAG)	1.76E-06	

(c) 2000-2014 Ingenuity Systems, Inc. All rights reserved.

INGENUITY® SYSTEMS

Top Diseases and Bio Functions

Diseases and Disorders

Name	p-value	# Molecules
Cancer	1.12E-05 - 7.71E-03	257
Cardiovascular Disease	3.08E-05 - 5.43E-03	69
Organismal Injury and Abnormalities	3.59E-05 - 6.77E-03	68
Dermatological Diseases and Conditions	7.69E-05 - 6.02E-03	36
Developmental Disorder	7.69E-05 - 8.30E-03	87

Molecular and Cellular Functions

Name	p-value	# Molecules
Protein Synthesis	2.49E-07 - 6.19E-04	72
Cellular Assembly and Organization	7.39E-07 - 7.42E-03	127
Cellular Function and Maintenance	7.39E-07 - 7.42E-03	122
Cell Death and Survival	1.04E-06 - 7.27E-03	207
Cellular Growth and Proliferation	2.23E-06 - 7.80E-03	222

Physiological System Development and Function

Name	p-value	# Molecules
Tissue Morphology	1.37E-07 - 8.30E-03	162
Organismal Survival	1.34E-06 - 7.41E-03	122
Connective Tissue Development and Function	1.37E-05 - 8.15E-03	94
Hematological System Development and Function	1.55E-05 - 6.74E-03	93
Hematopoiesis	1.55E-05 - 6.74E-03	51

(c) 2000-2014 Ingenuity Systems, Inc. All rights reserved.

Top Tox Functions

Assays: Clinical Chemistry and Hematology

Name	p-value	# Molecules
Increased Levels of Albumin	7.85E-02 - 7.85E-02	1
Increased Levels of Hematocrit	9.43E-02 - 9.43E-02	7
Increased Levels of Red Blood Cells	1.81E-01 - 1.81E-01	6
Decreased Levels of Albumin	2.18E-01 - 2.18E-01	1
Increased Levels of LDH	2.49E-01 - 2.49E-01	1

Cardiotoxicity

Name	p-value	# Molecules
Cardiac Arrythmia	5.43E-03 - 5.77E-01	10
Cardiac Proliferation	6.73E-03 - 3.82E-02	8
Bradycardia	2.94E-02 - 1.85E-01	4
Cardiac Inflammation	4.01E-02 - 5.01E-01	5
Congenital Heart Anomaly	4.01E-02 - 4.80E-01	9

Hepatotoxicity

Name	p-value	# Molecules
Liver Enlargement	1.60E-03 - 1.60E-03	2
Liver Necrosis/Cell Death	1.60E-03 - 1.00E00	11
Liver Proliferation	4.99E-03 - 4.36E-01	13
Liver Dysplasia	2.16E-02 - 7.85E-02	2
Liver Steatosis	3.76E-02 - 1.00E00	15

(c) 2000-2014 Ingenuity Systems, Inc. All rights reserved.

INGENUITY® SYSTEMS

Nephrotoxicity

Name	p-value	# Molecules
Renal Necrosis/Cell Death	1.99E-03 - 5.94E-01	26
Renal Proliferation	4.28E-03 - 5.06E-01	15
Nephrosis	4.01E-02 - 3.02E-01	3
Renal Inflammation	4.01E-02 - 1.00E00	7
Renal Nephritis	4.01E-02 - 1.00E00	7

Top Regulator Effect Networks

Top Networks

ID	Associated Network Functions	Score
1	Cellular Assembly and Organization, Cellular Function and Maintenance, Hematological System Development and Function	50
2	Cell Signaling, Cellular Assembly and Organization, DNA Replication, Recombination, and Repair	41
3	Lipid Metabolism, Molecular Transport, Small Molecule Biochemistry	39
4	Cell Death and Survival, Hematological System Development and Function, Immune Cell Trafficking	34
5	Cell Morphology, Nervous System Development and Function, Cell-To-Cell Signaling and Interaction	32

Top Tox Lists

Name	p-value	Ratio
Renal Ischemic Resistance Panel (Rat)	6.22E-03	3/10 (0.3)
Hypoxia-Inducible Factor Signaling	6.73E-03	8/70 (0.114)

(c) 2000-2014 Ingenuity Systems, Inc. All rights reserved.

INGENUITY® SYSTEMS

p53 Signaling	1.38E-02	9/95 (0.095)
Genes Upregulated in Response to Chronic Renal Failure (Rat)	1.48E-02	2/5 (0.4)
Cytochrome P450 Panel - Substrate is a Xenobiotic (Mouse)	1.65E-02	4/25 (0.16)

(c) 2000-2014 Ingenuity Systems, Inc. All rights reserved.

INGENUITY® SYSTEMS

Top My Lists

Name	p-value	Ratio

Top My Pathways

Name	p-value	Ratio

Top Molecules

This analysis has no expression values.

(c) 2000-2014 Ingenuity Systems, Inc. All rights reserved.
