# Supplementary material for: Systems biology surveillance decrypts pathological transcriptome remodeling
Source: BMC Syst Biol. 2015 Jul 17;9:36. doi: 10.1186/s12918-015-0177-8 (PMC4504166; doi:10.1186/s12918-015-0177-8)
Supplement: Additional file 1: — Functional enrichment data. Clustering Data: Provided are signaling pathways and gene networks enriched in each cluster, as well as gene IDs for all transcripts identified in the UMatrix analysis. Gene Ontology Data: Summarization of over represented functional themes in down and up regulated sub-transcriptomes for each of the truncation variants. [file 12918_2015_177_MOESM1_ESM.zip › 9929599221407335_add2.pdf]

IPA® Interactive pathway analysis of complex 'omics data

INGENUITY® SYSTEMS

Analysis Name: Cluster 2 - 2014-06-04 08:06 PM
Analysis Creation Date: 2014-06-04
Build version: 308606M
Content version: 18488943 (Release Date: 2014-03-23)

Analysis settings

View

Reference set: Mouse Genome 430 2.0 Array

Relationship to include: Direct and Indirect

Includes Endogenous Chemicals

Optional Analyses: My Pathways My List

Filter Summary:

Consider only relationships where

confidence = Experimentally Observed

Cutoff:

(c) 2000-2014 Ingenuity Systems, Inc. All rights reserved.

Top Canonical Pathways

Name	p-value	Ratio
RhoA Signaling	2.15E-03	8/128 (0.062)
Dendritic Cell Maturation	2.34E-03	9/211 (0.043)
Activation of IRF by Cytosolic Pattern Recognition Receptors	2.55E-03	5/73 (0.068)
Hypoxia Signaling in the Cardiovascular System	6.15E-03	5/68 (0.074)
Estrogen-Dependent Breast Cancer Signaling	6.6E-03	5/73 (0.068)

Top Upstream Regulators

Upstream Regulator	p-value of overlap	Predicted Activation State
palmitic acid	2.07E-05	
HNRNPC	5.95E-05	
NODAL	6.26E-04	
LRRK2	9.94E-04	
RPL5	9.94E-04	

(c) 2000-2014 Ingenuity Systems, Inc. All rights reserved.

Top Diseases and Bio Functions

Diseases and Disorders

Name	p-value	# Molecules
Cancer	1.75E-05 - 2.00E-02	237
Gastrointestinal Disease	1.75E-05 - 2.00E-02	103
Inflammatory Response	9.29E-04 - 2.08E-02	21
Neurological Disease	1.12E-03 - 1.95E-02	25
Connective Tissue Disorders	1.81E-03 - 1.95E-02	13

Molecular and Cellular Functions

Name	p-value	# Molecules
Lipid Metabolism	1.40E-04 - 2.01E-02	17
Small Molecule Biochemistry	1.40E-04 - 2.01E-02	25
Cell Cycle	3.78E-04 - 2.05E-02	26
Cell Morphology	3.78E-04 - 1.95E-02	49
Cellular Assembly and Organization	3.78E-04 - 2.19E-02	33

Physiological System Development and Function

Name	p-value	# Molecules
Nervous System Development and Function	3.78E-04 - 2.19E-02	29
Tumor Morphology	3.99E-04 - 1.95E-02	12
Hematological System Development and Function	4.55E-04 - 2.11E-02	32
Tissue Morphology	4.55E-04 - 1.95E-02	36
Lymphoid Tissue Structure and Development	4.96E-04 - 1.95E-02	22

(c) 2000-2014 Ingenuity Systems, Inc. All rights reserved.

INGENUITY® SYSTEMS

Top Tox Functions

Assays: Clinical Chemistry and Hematology

Name	p-value	# Molecules
Increased Levels of Alkaline Phosphatase	3.76E-01 - 3.76E-01	2

Cardiotoxicity

Name	p-value	# Molecules
Cardiac Hypertrophy	1.53E-02 - 4.04E-01	11
Cardiac Fibrosis	1.95E-02 - 3.86E-02	1
Cardiac Regeneration	3.86E-02 - 3.86E-02	1
Heart Failure	3.86E-02 - 5.72E-01	5
Cardiac Stress Response	5.73E-02 - 5.73E-02	1

Hepatotoxicity

Name	p-value	# Molecules
Liver Hyperplasia/Hyperproliferation	1.95E-02 - 1.00E00	10
Liver Steatosis	3.27E-02 - 5.45E-01	10
Biliary Hyperplasia	3.86E-02 - 3.86E-02	1
Liver Inflammation/Hepatitis	3.86E-02 - 5.45E-01	6
Hepatocellular Carcinoma	7.87E-02 - 1.00E00	7

Nephrotoxicity

Name	p-value	# Molecules
Renal Dysplasia	1.95E-02 - 1.95E-02	1
Renal Hypoplasia	1.95E-02 - 1.95E-02	1
Renal Inflammation	1.11E-01 - 1.00E00	4
Renal Nephritis	1.11E-01 - 1.00E00	4

(c) 2000-2014 Ingenuity Systems, Inc. All rights reserved.

Glomerular Injury	2.98E-01 - 3.64E-01	2

Top Regulator Effect Networks

Top Networks

ID	Associated Network Functions	Score
1	Cellular Function and Maintenance, Cellular Movement, Nervous System Development and Function	48
2	Cancer, Neurological Disease, Developmental Disorder	40
3	Humoral Immune Response, Protein Synthesis, Cell Morphology	38
4	Organ Morphology, Skeletal and Muscular System Development and Function, Cellular Movement	37
5	Gene Expression, Protein Synthesis, Cell Morphology	28

Top Tox Lists

Name	p-value	Ratio
Reversible Glomerulonephritis Biomarker Panel (Rat)	6.76E-02	2/27 (0.074)
Increases Heart Failure	7.31E-02	2/23 (0.087)
Cardiac Hypertrophy	8.37E-02	11/373 (0.029)
p53 Signaling	1.05E-01	4/99 (0.04)
Anti-Apoptosis	1.15E-01	2/32 (0.062)

(c) 2000-2014 Ingenuity Systems, Inc. All rights reserved.

INGENUITY® SYSTEMS

Top My Lists

Name	p-value	Ratio

Top My Pathways

Name	p-value	Ratio

Top Molecules

This analysis has no expression values.

(c) 2000-2014 Ingenuity Systems, Inc. All rights reserved.

INGENUITY® SYSTEMS
