# Supplementary material for: Systems biology surveillance decrypts pathological transcriptome remodeling
Source: BMC Syst Biol. 2015 Jul 17;9:36. doi: 10.1186/s12918-015-0177-8 (PMC4504166; doi:10.1186/s12918-015-0177-8)
Supplement: Additional file 1: — Functional enrichment data. Clustering Data: Provided are signaling pathways and gene networks enriched in each cluster, as well as gene IDs for all transcripts identified in the UMatrix analysis. Gene Ontology Data: Summarization of over represented functional themes in down and up regulated sub-transcriptomes for each of the truncation variants. [file 12918_2015_177_MOESM1_ESM.zip › 9929599221407335_add20.pdf]

Analysis Name: UP - FC (abs) ([NP] vs [Contr- 2013-02-28 01:18 PM
Analysis Creation Date: 2013-02-28
Build version: 302937
Content version: Not available.

Note: Since this analysis was run, curated information for 3% of the molecules has changed.
For the latest information, please run a new analysis on this dataset.

Analysis settings

View
Reference set: Ingenuity Knowledge Base (Genes Only)
Relationship to include: Direct and Indirect
Includes Endogenous Chemicals
Optional Analyses: My Pathways My List

Filter Summary:
Consider only relationships where
data sources = An Open Access Database of Genome-wide Association Results OR BIND OR BIOGRID OR Breast Cancer Information Core (BIC) OR Catalogue Of Somatic Mutations In Cancer (COSMIC) OR Chemical Carcinogenesis Research Information System (CCRIS) OR ClinicalTrials.gov OR ClinVar OR Cognia OR DIP OR DrugBank OR Gene Ontology (GO) OR GVK Biosciences OR Hazardous Substances Data Bank (HSDB) OR HumanCyc OR Ingenuity Expert Findings OR Ingenuity ExpertAssist Findings OR INTACT OR Interactome studies OR MINT OR MIPS OR miRBase OR miRecords OR Mouse Genome Database (MGD) OR Obesity Gene Map Database OR Online Mendelian Inheritance in Man (OMIM) OR TarBase OR TargetScan Human
Cutoff:

(c) 2000-2014 Ingenuity Systems, Inc. All rights reserved.

Top Canonical Pathways

Name	p-value	Ratio
ATM Signaling	2.74E-05	14/61 (0.23)
p53 Signaling	4.43E-04	16/96 (0.167)
Role of CHK Proteins in Cell Cycle Checkpoint Control	9.41E-04	11/57 (0.193)
Prostate Cancer Signaling	1.07E-03	14/98 (0.143)
Melanoma Signaling	1.62E-03	9/46 (0.196)

Top Upstream Regulators

Upstream Regulator	p-value of overlap	Predicted Activation State
miR-590-3p (miRNAs w/seed AAUUUUA)	1.01E-28	
miR-381-3p (and other miRNAs w/seed AUACAAG)	3.98E-27	
miR-340-5p (miRNAs w/seed UAUAAAG)	1.88E-26	
miR-144-3p (miRNAs w/seed ACAGUAU)	5.66E-24	
miR-30c-5p (and other miRNAs w/seed GUAAACA)	2.14E-22	

(c) 2000-2014 Ingenuity Systems, Inc. All rights reserved.

Top Diseases and Bio Functions

Diseases and Disorders

Name	p-value	# Molecules
Cancer	7.95E-06 - 1.30E-02	442
Gastrointestinal Disease	7.95E-06 - 1.30E-02	37
Infectious Disease	1.67E-04 - 1.30E-02	168
Reproductive System Disease	4.27E-04 - 1.13E-02	42
Neurological Disease	1.01E-03 - 1.30E-02	135

Molecular and Cellular Functions

Name	p-value	# Molecules
Cellular Growth and Proliferation	2.97E-08 - 1.30E-02	363
Gene Expression	2.40E-07 - 8.01E-03	255
Cell Death and Survival	4.25E-07 - 1.30E-02	362
RNA Post-Transcriptional Modification	5.64E-07 - 1.33E-03	44
Molecular Transport	6.27E-07 - 1.30E-02	176

Physiological System Development and Function

Name	p-value	# Molecules
Cardiovascular System Development and Function	1.28E-06 - 8.93E-03	135
Organ Morphology	1.28E-06 - 1.23E-02	121
Renal and Urological System Development and Function	3.16E-05 - 1.30E-02	16
Hair and Skin Development and Function	5.61E-05 - 1.30E-02	36
Connective Tissue Development and Function	2.15E-04 - 1.23E-02	58

(c) 2000-2014 Ingenuity Systems, Inc. All rights reserved.

INGENUITY SYSTEMS

Top Tox Functions

Assays: Clinical Chemistry and Hematology

Name	p-value	# Molecules
Decreased Levels of Potassium	2.48E-02 - 2.48E-02	2
Increased Levels of Red Blood Cells	1.06E-01 - 2.43E-01	10
Increased Levels of Bilirubin	1.30E-01 - 1.30E-01	1
Increased Levels of AST	2.43E-01 - 2.43E-01	1
Increased Levels of ALT	2.94E-01 - 2.94E-01	1

Cardiotoxicity

Name	p-value	# Molecules
Cardiac Fibrosis	1.30E-02 - 4.18E-01	4
Cardiac Necrosis/Cell Death	3.20E-02 - 1.00E00	20
Cardiac Hypertrophy	3.34E-02 - 4.19E-01	26
Cardiac Enlargement	3.95E-02 - 2.43E-01	3
Cardiac Damage	6.73E-02 - 4.66E-01	4

Hepatotoxicity

Name	p-value	# Molecules
Liver Hyperplasia/Hyperproliferation	1.53E-02 - 1.00E00	45
Liver Necrosis/Cell Death	2.70E-02 - 1.88E-01	21
Liver Proliferation	2.92E-02 - 3.72E-01	18
Liver Steatosis	2.98E-02 - 4.65E-01	26
Liver Hepatitis	3.55E-02 - 1.00E00	11

(c) 2000-2014 Ingenuity Systems, Inc. All rights reserved.

INGENUITY® SYSTEMS

Nephrotoxicity

Name	p-value	# Molecules
Glomerular Injury	9.07E-03 - 5.02E-01	9
Renal Hypertrophy	2.55E-02 - 2.55E-02	3
Renal Necrosis/Cell Death	3.77E-02 - 3.69E-01	40
Nephrosis	6.73E-02 - 5.96E-01	4
Renal Destruction	6.73E-02 - 6.73E-02	1

Top Regulator Effect Networks

Top Networks

ID	Associated Network Functions	Score
1	Hematological Disease, Cell Death and Survival, Cellular Development	48
2	Cellular Development, Hematological System Development and Function, Hematopoiesis	45
3	RNA Post-Transcriptional Modification, Molecular Transport, RNA Trafficking	42
4	Cellular Assembly and Organization, Molecular Transport, RNA Trafficking	42
5	Lipid Metabolism, Small Molecule Biochemistry, Embryonic Development	42

Top Tox Lists

Name	p-value	Ratio
p53 Signaling	5.65E-04	16/95 (0.168)
Cell Cycle: G1/S Checkpoint Regulation	2.44E-02	9/63 (0.143)

(c) 2000-2014 Ingenuity Systems, Inc. All rights reserved.

INGENUITY® SYSTEMS

Genes Upregulated in Response to Proteinuria-induced Oxidative Stress in Renal Proximal Tubule Cells (Human)	2.55E-02	3/10 (0.3)
Renal Necrosis/Cell Death	2.94E-02	40/437 (0.092)
RAR Activation	8.13E-02	17/175 (0.097)

(c) 2000-2014 Ingenuity Systems, Inc. All rights reserved.

Top My Lists

Name	p-value	Ratio

Top My Pathways

Name	p-value	Ratio

Top Molecules

This analysis has no expression values.

(c) 2000-2014 Ingenuity Systems, Inc. All rights reserved.
