# Supplementary material for: Systems biology surveillance decrypts pathological transcriptome remodeling
Source: BMC Syst Biol. 2015 Jul 17;9:36. doi: 10.1186/s12918-015-0177-8 (PMC4504166; doi:10.1186/s12918-015-0177-8)
Supplement: Additional file 1: — Functional enrichment data. Clustering Data: Provided are signaling pathways and gene networks enriched in each cluster, as well as gene IDs for all transcripts identified in the UMatrix analysis. Gene Ontology Data: Summarization of over represented functional themes in down and up regulated sub-transcriptomes for each of the truncation variants. [file 12918_2015_177_MOESM1_ESM.zip › 9929599221407335_add21.pdf]

Analysis Name: UP - FC (abs) ([PC] vs [Contr- 2013-02-28 01:20 PM
Analysis Creation Date: 2013-02-28
Build version: 302937
Content version: Not available.

Note: Since this analysis was run, curated information for 3% of the molecules has changed.
For the latest information, please run a new analysis on this dataset.

Analysis settings

View

Reference set: Ingenuity Knowledge Base (Genes Only)

Relationship to include: Direct and Indirect

Includes Endogenous Chemicals

Optional Analyses: My Pathways My List

Filter Summary:

Consider only relationships where

data sources = An Open Access Database of Genome-wide Association Results OR BIND OR BIOGRID OR Breast Cancer Information Core (BIC) OR Catalogue Of Somatic Mutations In Cancer (COSMIC) OR Chemical Carcinogenesis Research Information System (CCRIS) OR ClinicalTrials.gov OR ClinVar OR Cognia OR DIP OR DrugBank OR Gene Ontology (GO) OR GVK Biosciences OR Hazardous Substances Data Bank (HSDB) OR HumanCyc OR Ingenuity Expert Findings OR Ingenuity ExpertAssist Findings OR INTACT OR Interactome studies OR MINT OR MIPS OR miRBase OR miRecords OR Mouse Genome Database (MGD) OR Obesity Gene Map Database OR Online Mendelian Inheritance in Man (OMIM) OR TarBase OR TargetScan Human

Cutoff:

(c) 2000-2014 Ingenuity Systems, Inc. All rights reserved.

Top Canonical Pathways

Name	p-value	Ratio
ATM Signaling	4.64E-05	14/61 (0.23)
Role of CHK Proteins in Cell Cycle Checkpoint Control	3.71E-04	12/57 (0.211)
p53 Signaling	7.44E-04	16/96 (0.167)
Nur77 Signaling in T Lymphocytes	1.39E-03	11/63 (0.175)
B Cell Receptor Signaling	1.68E-03	22/170 (0.129)

Top Upstream Regulators

Upstream Regulator	p-value of overlap	Predicted Activation State
miR-590-3p (miRNAs w/seed AAUUUUA)	1.92E-29	
miR-381-3p (and other miRNAs w/seed AUACAAG)	7.77E-27	
miR-340-5p (miRNAs w/seed UAUAAAG)	4.42E-24	
miR-144-3p (miRNAs w/seed ACAGUAU)	6.34E-22	
miR-30c-5p (and other miRNAs w/seed GUAAACA)	1.42E-21	

(c) 2000-2014 Ingenuity Systems, Inc. All rights reserved.

INGENUITY® SYSTEMS

Top Diseases and Bio Functions

Diseases and Disorders

Name	p-value	# Molecules
Cancer	1.60E-05 - 1.42E-02	456
Gastrointestinal Disease	1.60E-05 - 1.42E-02	36
Infectious Disease	6.41E-05 - 1.37E-02	180
Neurological Disease	2.68E-04 - 1.42E-02	159
Developmental Disorder	4.66E-04 - 1.42E-02	123

Molecular and Cellular Functions

Name	p-value	# Molecules
Gene Expression	5.73E-08 - 1.10E-02	274
RNA Post-Transcriptional Modification	3.28E-07 - 2.33E-03	46
Cellular Growth and Proliferation	6.43E-07 - 1.42E-02	370
Cell Death and Survival	1.15E-06 - 1.42E-02	370
Cell Cycle	2.38E-06 - 1.42E-02	224

Physiological System Development and Function

Name	p-value	# Molecules
Cardiovascular System Development and Function	5.64E-06 - 7.73E-03	142
Organ Morphology	5.64E-06 - 1.42E-02	149
Hair and Skin Development and Function	9.05E-06 - 1.42E-02	46
Renal and Urological System Development and Function	1.17E-04 - 1.10E-02	13
Tissue Morphology	2.06E-04 - 1.42E-02	212

(c) 2000-2014 Ingenuity Systems, Inc. All rights reserved.

INGENUITY® SYSTEMS

Top Tox Functions

Assays: Clinical Chemistry and Hematology

Name	p-value	# Molecules
Decreased Levels of Albumin	6.17E-02 - 6.17E-02	2
Increased Levels of Albumin	7.06E-02 - 4.43E-01	2
Increased Levels of Red Blood Cells	1.33E-01 - 2.54E-01	10
Increased Levels of Bilirubin	1.36E-01 - 1.36E-01	1
Increased Levels of AST	2.54E-01 - 2.54E-01	1

Cardiotoxicity

Name	p-value	# Molecules
Cardiac Fibrosis	1.42E-02 - 1.00E00	9
Cardiac Hypertrophy	3.77E-02 - 4.76E-01	30
Cardiac Necrosis/Cell Death	3.82E-02 - 5.78E-01	22
Cardiac Enlargement	4.32E-02 - 2.54E-01	3
Cardiac Arrythmia	6.17E-02 - 5.85E-01	8

Hepatotoxicity

Name	p-value	# Molecules
Liver Hyperplasia/Hyperproliferation	1.80E-02 - 1.00E00	47
Liver Necrosis/Cell Death	2.12E-02 - 1.36E-01	25
Liver Proliferation	3.41E-02 - 5.77E-01	20
Liver Hematopoiesis	3.77E-02 - 4.32E-02	3
Hepatocellular Carcinoma	4.18E-02 - 1.00E00	45

(c) 2000-2014 Ingenuity Systems, Inc. All rights reserved.

INGENUITY® SYSTEMS

Nephrotoxicity

Name	p-value	# Molecules
Glomerular Injury	1.10E-02 - 6.14E-01	8
Renal Necrosis/Cell Death	2.72E-02 - 5.57E-01	41
Renal Hypertrophy	2.89E-02 - 3.07E-01	4
Renal Inflammation	6.17E-02 - 1.00E00	14
Renal Nephritis	6.17E-02 - 1.00E00	14

Top Regulator Effect Networks

Top Networks

ID	Associated Network Functions	Score
1	Cellular Assembly and Organization, Molecular Transport, RNA Trafficking	46
2	RNA Post-Transcriptional Modification, Embryonic Development, Organismal Development	41
3	Cell Cycle, Cellular Development, Cell Morphology	37
4	Cellular Function and Maintenance, Cellular Movement, Nervous System Development and Function	37
5	Nervous System Development and Function, Cell Cycle, Digestive System Development and Function	37

Top Tox Lists

Name	p-value	Ratio
p53 Signaling	9.44E-04	16/95 (0.168)
Genes Upregulated in Response to Proteinuria-induced Oxidative Stress in Renal Proximal Tubule Cells (Human)	2.89E-02	3/10 (0.3)

(c) 2000-2014 Ingenuity Systems, Inc. All rights reserved.

INGENUITY® SYSTEMS

Renal Necrosis/Cell Death	3.79E-02	41/437 (0.094)
Liver Necrosis/Cell Death	5.75E-02	25/254 (0.098)
Cell Cycle: G1/S Checkpoint Regulation	7.45E-02	8/63 (0.127)

(c) 2000-2014 Ingenuity Systems, Inc. All rights reserved.

INGENUITY® SYSTEMS

Top My Lists

Name	p-value	Ratio

Top My Pathways

Name	p-value	Ratio

Top Molecules

This analysis has no expression values.

(c) 2000-2014 Ingenuity Systems, Inc. All rights reserved.

INGENUITY® SYSTEMS
