# Supplementary material for: Systems biology surveillance decrypts pathological transcriptome remodeling
Source: BMC Syst Biol. 2015 Jul 17;9:36. doi: 10.1186/s12918-015-0177-8 (PMC4504166; doi:10.1186/s12918-015-0177-8)
Supplement: Additional file 1: — Functional enrichment data. Clustering Data: Provided are signaling pathways and gene networks enriched in each cluster, as well as gene IDs for all transcripts identified in the UMatrix analysis. Gene Ontology Data: Summarization of over represented functional themes in down and up regulated sub-transcriptomes for each of the truncation variants. [file 12918_2015_177_MOESM1_ESM.zip › 9929599221407335_add3.pdf]

Analysis Name: Cluster 3 - 2014-06-04 08:10 PM
Analysis Creation Date: 2014-06-04
Build version: 308606M
Content version: 18488943 (Release Date: 2014-03-23)

Analysis settings

View

Reference set: Mouse Genome 430 2.0 Array

Relationship to include: Direct and Indirect

Includes Endogenous Chemicals

Optional Analyses: My Pathways My List

Filter Summary:

Consider only relationships where

confidence = Experimentally Observed

Cutoff:

(c) 2000-2014 Ingenuity Systems, Inc. All rights reserved.

Top Canonical Pathways

Name	p-value	Ratio
EIF2 Signaling	2.03E-06	6/201 (0.03)
Regulation of eIF4 and p70S6K Signaling	3.98E-03	3/175 (0.017)
Prostanoid Biosynthesis	2.2E-02	1/15 (0.067)
Antiproliferative Role of TOB in T Cell Signaling	6.23E-02	1/26 (0.038)
mTOR Signaling	6.64E-02	2/213 (0.009)

Top Upstream Regulators

Upstream Regulator	p-value of overlap	Predicted Activation State
FAAH	4.59E-05	
CD 437	1.18E-03	
MYCN	2.05E-03	
Epbp	2.51E-03	
NYX	2.51E-03	

(c) 2000-2014 Ingenuity Systems, Inc. All rights reserved.

Top Diseases and Bio Functions

Diseases and Disorders

Name	p-value	# Molecules
Connective Tissue Disorders	2.47E-03 - 1.47E-02	2
Developmental Disorder	2.47E-03 - 3.16E-02	4
Hematological Disease	2.47E-03 - 4.35E-02	3
Hereditary Disorder	2.47E-03 - 2.92E-02	6
Neurological Disease	2.47E-03 - 4.93E-03	1

Molecular and Cellular Functions

Name	p-value	# Molecules
Cell Cycle	2.47E-03 - 3.40E-02	4
Cell Death and Survival	2.47E-03 - 3.69E-02	3
Cellular Development	2.47E-03 - 4.59E-02	5
Cellular Growth and Proliferation	2.47E-03 - 4.59E-02	3
Lipid Metabolism	2.47E-03 - 4.59E-02	1

Physiological System Development and Function

Name	p-value	# Molecules
Behavior	2.47E-03 - 2.47E-03	1
Connective Tissue Development and Function	2.47E-03 - 4.35E-02	3
Hematological System Development and Function	2.47E-03 - 3.88E-02	4
Hematopoiesis	2.47E-03 - 3.64E-02	2
Humoral Immune Response	2.47E-03 - 3.40E-02	1

(c) 2000-2014 Ingenuity Systems, Inc. All rights reserved.

INGENUITY SYSTEMS

Top Tox Functions

Assays: Clinical Chemistry and Hematology

Name	p-value	# Molecules
Decreased Levels of Potassium	9.83E-03 - 9.83E-03	1

Cardiotoxicity

Name	p-value	# Molecules
Cardiac Dysfunction	7.38E-03 - 7.38E-03	1
Cardiac Dilation	9.64E-02 - 9.64E-02	1
Cardiac Fibrosis	2.31E-01 - 2.31E-01	1
Cardiac Hypertrophy	2.54E-01 - 2.54E-01	1

Hepatotoxicity

Name	p-value	# Molecules
Liver Steatosis	4.80E-01 - 4.80E-01	1
Liver Hyperplasia/Hyperproliferation	1.00E00 - 1.00E00	1

Nephrotoxicity

Name	p-value	# Molecules
Renal Destruction	2.47E-03 - 2.47E-03	1
Renal Proliferation	3.36E-01 - 3.36E-01	1
Renal Necrosis/Cell Death	4.62E-01 - 4.62E-01	1

Top Regulator Effect Networks

(c) 2000-2014 Ingenuity Systems, Inc. All rights reserved.

INGENUITY® SYSTEMS

Top Networks

ID	Associated Network Functions	Score
1	Hereditary Disorder, Ophthalmic Disease, Cellular Assembly and Organization	38
2	Endocrine System Disorders, Gastrointestinal Disease, Metabolic Disease	36
3	Lipid Metabolism, Small Molecule Biochemistry, Cardiovascular System Development and Function	18

Top Tox Lists

Name	p-value	Ratio
Decreases Transmembrane Potential of Mitochondria and Mitochondrial Membrane	2.25E-01	1/117 (0.009)
Increases Renal Proliferation	2.61E-01	1/128 (0.008)
Mitochondrial Dysfunction	3.04E-01	1/169 (0.006)
Cardiac Fibrosis	3.41E-01	1/181 (0.006)
Cardiac Hypertrophy	5.85E-01	1/373 (0.003)

(c) 2000-2014 Ingenuity Systems, Inc. All rights reserved.

Top My Lists

Name	p-value	Ratio

Top My Pathways

Name	p-value	Ratio

Top Molecules

This analysis has no expression values.

(c) 2000-2014 Ingenuity Systems, Inc. All rights reserved.
