# Supplementary material for: Systems biology surveillance decrypts pathological transcriptome remodeling
Source: BMC Syst Biol. 2015 Jul 17;9:36. doi: 10.1186/s12918-015-0177-8 (PMC4504166; doi:10.1186/s12918-015-0177-8)
Supplement: Additional file 1: — Functional enrichment data. Clustering Data: Provided are signaling pathways and gene networks enriched in each cluster, as well as gene IDs for all transcripts identified in the UMatrix analysis. Gene Ontology Data: Summarization of over represented functional themes in down and up regulated sub-transcriptomes for each of the truncation variants. [file 12918_2015_177_MOESM1_ESM.zip › 9929599221407335_add4.pdf]

Analysis Name: Cluster 4 - 2014-06-04 08:11 PM
Analysis Creation Date: 2014-06-04
Build version: 308606M
Content version: 18488943 (Release Date: 2014-03-23)

Analysis settings

View
Reference set: Mouse Genome 430 2.0 Array
Relationship to include: Direct and Indirect
Includes Endogenous Chemicals
Optional Analyses: My Pathways My List

Filter Summary:
Consider only relationships where
confidence = Experimentally Observed
Cutoff:

(c) 2000-2014 Ingenuity Systems, Inc. All rights reserved.

Top Canonical Pathways

Name	p-value	Ratio
Hepatic Cholestasis	2.14E-03	12/183 (0.066)
Oxidative Phosphorylation	4.38E-03	9/120 (0.075)
OX40 Signaling Pathway	5.85E-03	6/97 (0.062)
Glycerol Degradation I	1.15E-02	2/12 (0.167)
BMP signaling pathway	1.23E-02	7/86 (0.081)

Top Upstream Regulators

Upstream Regulator	p-value of overlap	Predicted Activation State
Mup1 (includes others)	9.86E-05	
KAT2A	1.72E-04	
RAR ligand-RARα-Retinoic acid-RXRα	1.74E-04	
Rxr	1.95E-04	
PRKAR1A	2.46E-04	

(c) 2000-2014 Ingenuity Systems, Inc. All rights reserved.

INGENUITY® SYSTEMS

Top Diseases and Bio Functions

Diseases and Disorders

Name	p-value	# Molecules
Dermatological Diseases and Conditions	5.36E-04 - 3.52E-02	52
Developmental Disorder	5.36E-04 - 3.52E-02	47
Hereditary Disorder	5.36E-04 - 3.52E-02	70
Organismal Injury and Abnormalities	5.36E-04 - 3.52E-02	171
Inflammatory Response	8.03E-04 - 3.52E-02	25

Molecular and Cellular Functions

Name	p-value	# Molecules
Cellular Development	9.52E-05 - 3.52E-02	108
Cellular Growth and Proliferation	9.52E-05 - 3.36E-02	51
Lipid Metabolism	1.12E-04 - 3.52E-02	10
Molecular Transport	1.12E-04 - 3.52E-02	42
Small Molecule Biochemistry	1.12E-04 - 3.52E-02	26

Physiological System Development and Function

Name	p-value	# Molecules
Hematological System Development and Function	9.52E-05 - 3.52E-02	50
Digestive System Development and Function	5.49E-04 - 3.52E-02	31
Embryonic Development	5.49E-04 - 3.52E-02	67
Organ Development	5.49E-04 - 3.52E-02	43
Organismal Development	5.49E-04 - 3.52E-02	79

(c) 2000-2014 Ingenuity Systems, Inc. All rights reserved.

INGENUITY® SYSTEMS

Top Tox Functions

Assays: Clinical Chemistry and Hematology

Name	p-value	# Molecules
Increased Levels of CRP	3.52E-02 - 3.52E-02	1
Increased Levels of Blood Urea Nitrogen	8.52E-02 - 8.52E-02	2
Increased Levels of ALT	1.64E-01 - 1.64E-01	1
Increased Levels of Bilirubin	1.64E-01 - 1.64E-01	1
Increased Levels of AST	1.94E-01 - 1.94E-01	1

Cardiotoxicity

Name	p-value	# Molecules
Cardiac Arrythmia	4.88E-03 - 5.41E-01	9
Cardiac Arteriopathy	9.55E-03 - 9.88E-02	14
Bradycardia	2.31E-02 - 2.31E-02	2
Pulmonary Hypertension	3.52E-02 - 3.52E-02	1
Tachycardia	3.52E-02 - 5.41E-01	6

Hepatotoxicity

Name	p-value	# Molecules
Liver Damage	7.91E-03 - 1.00E00	5
Liver Cholestasis	1.31E-02 - 5.51E-01	8
Liver Inflammation/Hepatitis	1.69E-02 - 4.05E-01	10
Hepatocellular Carcinoma	3.52E-02 - 5.77E-01	20
Liver Hyperbilirubinemia	3.52E-02 - 3.52E-02	1

Nephrotoxicity

(c) 2000-2014 Ingenuity Systems, Inc. All rights reserved.

INGENUITY® SYSTEMS

Name	p-value	# Molecules
Renal Atrophy	3.61E-02 - 3.61E-02	3
Nephrosis	6.92E-02 - 3.73E-01	2
Renal Hypertrophy	6.92E-02 - 6.92E-02	1
Glomerular Injury	1.02E-01 - 5.30E-01	3
Kidney Failure	1.02E-01 - 5.30E-01	8

Top Regulator Effect Networks

Top Networks

ID	Associated Network Functions	Score
1	DNA Replication, Recombination, and Repair, Energy Production, Nucleic Acid Metabolism	38
2	Cell Signaling, Hereditary Disorder, Neurological Disease	36
3	Embryonic Development, Organismal Development, Tissue Development	34
4	Lipid Metabolism, Molecular Transport, Small Molecule Biochemistry	34
5	Cellular Development, Connective Tissue Disorders, Developmental Disorder	32

Top Tox Lists

Name	p-value	Ratio
Hepatic Cholestasis	2.59E-03	12/142 (0.085)
Genes Upregulated in Response to Chronic Renal Failure (Rat)	1.15E-02	2/5 (0.4)
Mitochondrial Dysfunction	1.42E-02	11/169 (0.065)
FXR/RXR Activation	2.39E-02	7/86 (0.081)
PPARα/RXRα Activation	3.83E-02	11/182 (0.06)

(c) 2000-2014 Ingenuity Systems, Inc. All rights reserved.

INGENUITY® SYSTEMS

Top My Lists

Name	p-value	Ratio

Top My Pathways

Name	p-value	Ratio

Top Molecules

This analysis has no expression values.

(c) 2000-2014 Ingenuity Systems, Inc. All rights reserved.
