# Supplementary material for: Systems biology surveillance decrypts pathological transcriptome remodeling
Source: BMC Syst Biol. 2015 Jul 17;9:36. doi: 10.1186/s12918-015-0177-8 (PMC4504166; doi:10.1186/s12918-015-0177-8)
Supplement: Additional file 1: — Functional enrichment data. Clustering Data: Provided are signaling pathways and gene networks enriched in each cluster, as well as gene IDs for all transcripts identified in the UMatrix analysis. Gene Ontology Data: Summarization of over represented functional themes in down and up regulated sub-transcriptomes for each of the truncation variants. [file 12918_2015_177_MOESM1_ESM.zip › 9929599221407335_add5.pdf]

Analysis Name: Cluster 5 - 2014-06-04 08:11 PM
Analysis Creation Date: 2014-06-04
Build version: 308606M
Content version: 18488943 (Release Date: 2014-03-23)

Analysis settings

View

Reference set: Mouse Genome 430 2.0 Array

Relationship to include: Direct and Indirect

Includes Endogenous Chemicals

Optional Analyses: My Pathways My List

Filter Summary:

Consider only relationships where

confidence = Experimentally Observed

Cutoff:

(c) 2000-2014 Ingenuity Systems, Inc. All rights reserved.

Top Canonical Pathways

Name	p-value	Ratio
Remodeling of Epithelial Adherens Junctions	1.88E-02	4/70 (0.057)
CDP-diacylglycerol Biosynthesis I	2.31E-02	2/27 (0.074)
Phosphatidylglycerol Biosynthesis II (Non-plastidic)	2.93E-02	2/33 (0.061)
Asparagine Degradation I	3.15E-02	1/5 (0.2)
Spermine Biosynthesis	3.15E-02	1/7 (0.143)

Top Upstream Regulators

Upstream Regulator	p-value of overlap	Predicted Activation State
WWP1	2.27E-04	
E2F4	2.47E-04	
Nr1h	4.49E-04	
PRKAG3	8.80E-04	
RORA	1.09E-03	

(c) 2000-2014 Ingenuity Systems, Inc. All rights reserved.

Top Diseases and Bio Functions

Diseases and Disorders

Name	p-value	# Molecules
Cancer	7.60E-07 - 4.63E-02	192
Organismal Injury and Abnormalities	1.57E-05 - 3.96E-02	95
Reproductive System Disease	1.57E-05 - 1.59E-02	83
Gastrointestinal Disease	7.00E-04 - 4.63E-02	90
Infectious Disease	1.15E-03 - 3.15E-02	9

Molecular and Cellular Functions

Name	p-value	# Molecules
Cell Cycle	3.01E-04 - 4.69E-02	32
Lipid Metabolism	7.47E-04 - 3.86E-02	24
Molecular Transport	7.47E-04 - 4.69E-02	28
Small Molecule Biochemistry	7.47E-04 - 4.69E-02	30
Cellular Assembly and Organization	7.85E-04 - 4.34E-02	34

Physiological System Development and Function

Name	p-value	# Molecules
Cardiovascular System Development and Function	3.58E-05 - 3.96E-02	22
Organ Morphology	3.58E-05 - 4.69E-02	20
Organismal Development	3.58E-05 - 4.69E-02	34
Hair and Skin Development and Function	3.01E-04 - 4.69E-02	12
Hematological System Development and Function	2.19E-03 - 4.69E-02	18

(c) 2000-2014 Ingenuity Systems, Inc. All rights reserved.

Top Tox Functions

Assays: Clinical Chemistry and Hematology

Name	p-value	# Molecules
Decreased Levels of Albumin	9.17E-02 - 9.17E-02	1
Increased Levels of Hematocrit	1.69E-01 - 1.69E-01	3
Increased Levels of Red Blood Cells	1.72E-01 - 1.72E-01	3
Increased Levels of Creatinine	2.01E-01 - 2.01E-01	1

Cardiotoxicity

Name	p-value	# Molecules
Cardiac Arrythmia	1.59E-02 - 1.00E00	3
Cardiac Arteriopathy	1.59E-02 - 6.83E-02	8
Cardiac Damage	1.59E-02 - 1.27E-01	2
Congenital Heart Anomaly	1.59E-02 - 5.37E-01	3
Cardiac Fibrosis	3.15E-02 - 5.05E-01	3

Hepatotoxicity

Name	p-value	# Molecules
Liver Cholestasis	5.34E-02 - 5.66E-01	4
Liver Dysplasia	9.17E-02 - 9.17E-02	1
Hepatocellular Carcinoma	1.06E-01 - 5.07E-01	8
Liver Hyperplasia/Hyperproliferation	1.06E-01 - 5.30E-01	12
Liver Inflammation/Hepatitis	3.20E-01 - 4.74E-01	2

Nephrotoxicity

Name	p-value	# Molecules
Nephrosis	1.59E-02 - 1.59E-02	1

(c) 2000-2014 Ingenuity Systems, Inc. All rights reserved.

INGENUITY® SYSTEMS

Renal Inflammation	1.06E-01 - 1.06E-01	1
Renal Nephritis	1.06E-01 - 1.06E-01	1
Renal Proliferation	1.23E-01 - 2.51E-01	6
Kidney Failure	1.48E-01 - 1.48E-01	1

Top Regulator Effect Networks

Top Networks

ID	Associated Network Functions	Score
1	Cellular Assembly and Organization, Developmental Disorder, Hereditary Disorder	64
2	Infectious Disease, Embryonic Development, Nervous System Development and Function	43
3	Cell Death and Survival, Cell Cycle, Cellular Movement	39
4	Lipid Metabolism, Molecular Transport, Small Molecule Biochemistry	34
5	RNA Post-Transcriptional Modification, Cancer, Cell-To-Cell Signaling and Interaction	24

Top Tox Lists

Name	p-value	Ratio
Genes Downregulated in Response to Chronic Renal Failure (Rat)	1.06E-01	1/10 (0.1)
Irreversible Glomerulonephritis Biomarker Panel (Rat)	1.34E-01	1/13 (0.077)
PPARα/RXRα Activation	1.35E-01	5/182 (0.027)
Renal Necrosis/Cell Death	1.78E-01	10/472 (0.021)
Cytochrome P450 Panel - Substrate is a Sterol (Rat)	1.88E-01	1/14 (0.071)

(c) 2000-2014 Ingenuity Systems, Inc. All rights reserved.

INGENUITY® SYSTEMS

Top My Lists

Name	p-value	Ratio

Top My Pathways

Name	p-value	Ratio

Top Molecules

This analysis has no expression values.

(c) 2000-2014 Ingenuity Systems, Inc. All rights reserved.
