# Supplementary material for: Systems biology surveillance decrypts pathological transcriptome remodeling
Source: BMC Syst Biol. 2015 Jul 17;9:36. doi: 10.1186/s12918-015-0177-8 (PMC4504166; doi:10.1186/s12918-015-0177-8)
Supplement: Additional file 1: — Functional enrichment data. Clustering Data: Provided are signaling pathways and gene networks enriched in each cluster, as well as gene IDs for all transcripts identified in the UMatrix analysis. Gene Ontology Data: Summarization of over represented functional themes in down and up regulated sub-transcriptomes for each of the truncation variants. [file 12918_2015_177_MOESM1_ESM.zip › 9929599221407335_add6.pdf]

Analysis Name: Cluster 6 - 2014-06-04 08:12 PM
Analysis Creation Date: 2014-06-04
Build version: 308606M
Content version: 18488943 (Release Date: 2014-03-23)

Analysis settings

View

Reference set: Mouse Genome 430 2.0 Array

Relationship to include: Direct and Indirect

Includes Endogenous Chemicals

Optional Analyses: My Pathways My List

Filter Summary:

Consider only relationships where

confidence = Experimentally Observed

Cutoff:

(c) 2000-2014 Ingenuity Systems, Inc. All rights reserved.

Top Canonical Pathways

Name	p-value	Ratio
Noradrenaline and Adrenaline Degradation	1.09E-03	5/53 (0.094)
Dopamine Degradation	2.13E-03	4/38 (0.105)
D-myo-inositol (1,4,5,6)-Tetrakisphosphate Biosynthesis	6.02E-03	9/144 (0.062)
D-myo-inositol (3,4,5,6)-tetrakisphosphate Biosynthesis	6.02E-03	9/144 (0.062)
Nur77 Signaling in T Lymphocytes	1.06E-02	5/64 (0.078)

Top Upstream Regulators

Upstream Regulator	p-value of overlap	Predicted Activation State
CLCF1	8.49E-04	
butylphen	2.50E-03	
3,3'-diindolylmethane	2.89E-03	
CLDN7	4.07E-03	
NLRC5	4.46E-03	

(c) 2000-2014 Ingenuity Systems, Inc. All rights reserved.

Top Diseases and Bio Functions

Diseases and Disorders

Name	p-value	# Molecules
Cancer	7.02E-04 - 3.42E-02	153
Organismal Injury and Abnormalities	7.02E-04 - 3.37E-02	68
Reproductive System Disease	7.02E-04 - 3.37E-02	45
Hematological Disease	1.79E-03 - 3.24E-02	23
Immunological Disease	1.79E-03 - 3.24E-02	17

Molecular and Cellular Functions

Name	p-value	# Molecules
Carbohydrate Metabolism	2.56E-05 - 3.01E-02	15
Small Molecule Biochemistry	2.56E-05 - 2.99E-02	31
Lipid Metabolism	1.04E-04 - 2.99E-02	14
Cell Death and Survival	7.43E-04 - 3.42E-02	29
Cellular Movement	8.48E-04 - 3.01E-02	12

Physiological System Development and Function

Name	p-value	# Molecules
Embryonic Development	2.53E-04 - 2.99E-02	40
Tissue Development	2.53E-04 - 3.08E-02	47
Tumor Morphology	7.43E-04 - 3.42E-02	26
Nervous System Development and Function	8.48E-04 - 3.42E-02	33
Organ Morphology	1.19E-03 - 2.99E-02	18

(c) 2000-2014 Ingenuity Systems, Inc. All rights reserved.

INGENUITY® SYSTEMS

Top Tox Functions

Assays: Clinical Chemistry and Hematology

Name	p-value	# Molecules
Increased Levels of Red Blood Cells	8.70E-02 - 8.70E-02	1
Decreased Levels of Hematocrit	2.16E-01 - 2.16E-01	1

Cardiotoxicity

Name	p-value	# Molecules
Cardiac Necrosis/Cell Death	2.99E-02 - 1.92E-01	2
Cardiac Arrythmia	5.89E-02 - 1.00E00	4
Tachycardia	5.89E-02 - 5.32E-01	3
Congenital Heart Anomaly	9.02E-02 - 1.00E00	3
Cardiac Enlargement	1.41E-01 - 1.41E-01	1

Hepatotoxicity

Name	p-value	# Molecules
Liver Fibrosis	7.14E-03 - 7.14E-03	9
Liver Necrosis/Cell Death	2.99E-02 - 3.60E-01	10
Biliary Hyperplasia	5.89E-02 - 5.89E-02	1
Liver Enlargement	5.89E-02 - 5.89E-02	1
Liver Hyperplasia/Hyperproliferation	5.89E-02 - 1.00E00	16

Nephrotoxicity

Name	p-value	# Molecules
Nephrosis	2.99E-02 - 2.99E-02	1
Renal Dysplasia	2.99E-02 - 3.85E-01	2
Glomerular Injury	3.42E-02 - 4.21E-01	7

(c) 2000-2014 Ingenuity Systems, Inc. All rights reserved.

INGENUITY® SYSTEMS

Renal Fibrosis	3.42E-02 - 4.06E-01	3
Kidney Failure	5.89E-02 - 1.00E00	10

Top Regulator Effect Networks

Top Networks

ID	Associated Network Functions	Score
1	Embryonic Development, Tissue Development, Gastrointestinal Disease	42
2	Cell Morphology, Cell-To-Cell Signaling and Interaction, Inflammatory Response	40
3	Carbohydrate Metabolism, Lipid Metabolism, Small Molecule Biochemistry	39
4	Cancer, Cellular Development, Cellular Growth and Proliferation	38
5	Nervous System Development and Function, Cardiovascular Disease, Cellular Assembly and Organization	35

Top Tox Lists

Name	p-value	Ratio
Genes Upregulated in Response to Proteinuria-induced Oxidative Stress in Renal Proximal Tubule Cells (Human)	1.95E-03	3/10 (0.3)
Xenobiotic Metabolism Signaling	8.31E-02	13/336 (0.039)
Cell Cycle: G1/S Checkpoint Regulation	1.14E-01	4/69 (0.058)
Decreases Transmembrane Potential of Mitochondria and Mitochondrial Membrane	1.95E-01	5/117 (0.043)
Increases Permeability Transition of Mitochondria and Mitochondrial Membrane	2.16E-01	1/8 (0.125)

(c) 2000-2014 Ingenuity Systems, Inc. All rights reserved.

INGENUITY® SYSTEMS

Top My Lists

Name	p-value	Ratio

Top My Pathways

Name	p-value	Ratio

Top Molecules

This analysis has no expression values.

(c) 2000-2014 Ingenuity Systems, Inc. All rights reserved.
