# Supplementary material for: Systems biology surveillance decrypts pathological transcriptome remodeling
Source: BMC Syst Biol. 2015 Jul 17;9:36. doi: 10.1186/s12918-015-0177-8 (PMC4504166; doi:10.1186/s12918-015-0177-8)
Supplement: Additional file 1: — Functional enrichment data. Clustering Data: Provided are signaling pathways and gene networks enriched in each cluster, as well as gene IDs for all transcripts identified in the UMatrix analysis. Gene Ontology Data: Summarization of over represented functional themes in down and up regulated sub-transcriptomes for each of the truncation variants. [file 12918_2015_177_MOESM1_ESM.zip › 9929599221407335_add7.pdf]

Analysis Name: Cluster 7 - 2014-06-04 08:13 PM
Analysis Creation Date: 2014-06-04
Build version: 308606M
Content version: 18488943 (Release Date: 2014-03-23)

Analysis settings

View

Reference set: Mouse Genome 430 2.0 Array

Relationship to include: Direct and Indirect

Includes Endogenous Chemicals

Optional Analyses: My Pathways My List

Filter Summary:

Consider only relationships where

confidence = Experimentally Observed

Cutoff:

(c) 2000-2014 Ingenuity Systems, Inc. All rights reserved.

Top Canonical Pathways

Name	p-value	Ratio
EIF2 Signaling	4.33E-05	5/201 (0.025)
Biotin-carboxyl Carrier Protein Assembly	1.01E-02	1/15 (0.067)
Ceramide Biosynthesis	1.51E-02	1/16 (0.062)
Tryptophan Degradation to 2-amino-3-carboxymuconate Semialdehyde	1.76E-02	1/18 (0.056)
Oxidative Phosphorylation	2.18E-02	2/120 (0.017)

Top Upstream Regulators

Upstream Regulator	p-value of overlap	Predicted Activation State
MYCN	2.46E-04	
CD 437	1.42E-03	
interferon beta-1a	3.01E-03	
cortisone	5.25E-03	
RFX4	5.25E-03	

(c) 2000-2014 Ingenuity Systems, Inc. All rights reserved.

INGENUITY® SYSTEMS

Top Diseases and Bio Functions

Diseases and Disorders

Name	p-value	# Molecules
Cancer	2.43E-03 - 4.71E-02	11
Organismal Injury and Abnormalities	2.43E-03 - 4.09E-02	9
Reproductive System Disease	2.43E-03 - 4.09E-02	7
Developmental Disorder	2.54E-03 - 2.54E-03	2
Hematological Disease	2.54E-03 - 5.07E-03	3

Molecular and Cellular Functions

Name	p-value	# Molecules
Cell Cycle	1.40E-03 - 3.74E-02	5
DNA Replication, Recombination, and Repair	1.40E-03 - 3.98E-02	4
Protein Synthesis	2.40E-03 - 4.71E-02	4
Amino Acid Metabolism	2.54E-03 - 3.00E-02	1
Cell Death and Survival	2.54E-03 - 4.71E-02	5

Physiological System Development and Function

Name	p-value	# Molecules
Hematological System Development and Function	2.54E-03 - 4.71E-02	2
Hematopoiesis	2.54E-03 - 2.54E-03	1
Skeletal and Muscular System Development and Function	2.54E-03 - 7.59E-03	2
Embryonic Development	5.07E-03 - 2.01E-02	2
Nervous System Development and Function	5.07E-03 - 3.98E-02	4

(c) 2000-2014 Ingenuity Systems, Inc. All rights reserved.

Top Tox Functions

Assays: Clinical Chemistry and Hematology

Name	p-value	# Molecules
Increased Levels of Hematocrit	2.03E-01 - 2.03E-01	1

Hepatotoxicity

Name	p-value	# Molecules
Liver Enlargement	5.07E-03 - 5.07E-03	1
Liver Necrosis/Cell Death	5.07E-03 - 1.37E-01	2
Liver Hyperplasia/Hyperproliferation	6.47E-02 - 1.37E-01	4
Hepatocellular Carcinoma	7.42E-02 - 7.42E-02	3
Liver Proliferation	3.89E-01 - 3.89E-01	1

Nephrotoxicity

Name	p-value	# Molecules
Renal Necrosis/Cell Death	3.98E-02 - 4.71E-01	2

Top Regulator Effect Networks

Top Networks

ID	Associated Network Functions	Score
1	Nucleic Acid Metabolism, Small Molecule Biochemistry, Developmental Disorder	39
2	Gene Expression, Protein Synthesis, Cell Death and Survival	29
3	Cell Cycle, Cell-To-Cell Signaling and Interaction, Cancer	14

(c) 2000-2014 Ingenuity Systems, Inc. All rights reserved.

INGENUITY® SYSTEMS

4	Carbohydrate Metabolism, Lipid Metabolism, Molecular Transport	3
5	Small Molecule Biochemistry, Organismal Development, Cancer	3

Top Tox Lists

Name	p-value	Ratio
Mitochondrial Dysfunction	5.27E-02	2/169 (0.012)
Pro-Apoptosis	9.44E-02	1/42 (0.024)
Cell Cycle: G2/M DNA Damage Checkpoint Regulation	1.08E-01	1/48 (0.021)
Liver Necrosis/Cell Death	1.41E-01	2/271 (0.007)
Decreases Transmembrane Potential of Mitochondria and Mitochondrial Membrane	2.31E-01	1/117 (0.009)

(c) 2000-2014 Ingenuity Systems, Inc. All rights reserved.

INGENUITY® SYSTEMS

Top My Lists

Name	p-value	Ratio

Top My Pathways

Name	p-value	Ratio

Top Molecules

This analysis has no expression values.

(c) 2000-2014 Ingenuity Systems, Inc. All rights reserved.

INGENUITY® SYSTEMS
