# Supplementary material for: Systems biology surveillance decrypts pathological transcriptome remodeling
Source: BMC Syst Biol. 2015 Jul 17;9:36. doi: 10.1186/s12918-015-0177-8 (PMC4504166; doi:10.1186/s12918-015-0177-8)
Supplement: Additional file 1: — Functional enrichment data. Clustering Data: Provided are signaling pathways and gene networks enriched in each cluster, as well as gene IDs for all transcripts identified in the UMatrix analysis. Gene Ontology Data: Summarization of over represented functional themes in down and up regulated sub-transcriptomes for each of the truncation variants. [file 12918_2015_177_MOESM1_ESM.zip › 9929599221407335_add8.pdf]

Analysis Name: Cluster 8 - 2014-06-04 08:14 PM
Analysis Creation Date: 2014-06-04
Build version: 308606M
Content version: 18488943 (Release Date: 2014-03-23)

Analysis settings

View

Reference set: Mouse Genome 430 2.0 Array

Relationship to include: Direct and Indirect

Includes Endogenous Chemicals

Optional Analyses: My Pathways My List

Filter Summary:

Consider only relationships where

confidence = Experimentally Observed

Cutoff:

(c) 2000-2014 Ingenuity Systems, Inc. All rights reserved.

Top Canonical Pathways

Name	p-value	Ratio
EIF2 Signaling	7.01E-05	16/201 (0.08)
Gαs Signaling	2.2E-03	10/125 (0.08)
cAMP-mediated signaling	3.06E-03	15/226 (0.066)
Regulation of eIF4 and p70S6K Signaling	1.21E-02	10/175 (0.057)
mTOR Signaling	1.26E-02	12/213 (0.056)

Top Upstream Regulators

Upstream Regulator	p-value of overlap	Predicted Activation State
MTA1	3.85E-04	
Histone h3	8.23E-04	
MITF	9.60E-04	
etorphine	1.07E-03	
HES5	1.11E-03	

(c) 2000-2014 Ingenuity Systems, Inc. All rights reserved.

INGENUITY® SYSTEMS

Top Diseases and Bio Functions

Diseases and Disorders

Name	p-value	# Molecules
Neurological Disease	1.45E-05 - 3.35E-02	30
Psychological Disorders	1.45E-05 - 3.35E-02	18
Hereditary Disorder	2.99E-05 - 3.35E-02	42
Ophthalmic Disease	2.99E-05 - 3.35E-02	26
Inflammatory Response	7.98E-05 - 3.35E-02	22

Molecular and Cellular Functions

Name	p-value	# Molecules
Cell Death and Survival	1.77E-05 - 3.35E-02	51
Protein Synthesis	1.99E-04 - 1.92E-02	53
Gene Expression	2.04E-04 - 2.87E-02	107
Cell-To-Cell Signaling and Interaction	4.95E-04 - 3.35E-02	66
Cellular Assembly and Organization	4.95E-04 - 3.35E-02	33

Physiological System Development and Function

Name	p-value	# Molecules
Embryonic Development	3.56E-04 - 3.35E-02	70
Organ Development	3.56E-04 - 3.35E-02	51
Organ Morphology	3.56E-04 - 3.35E-02	54
Organismal Development	3.56E-04 - 3.35E-02	87
Reproductive System Development and Function	3.56E-04 - 3.35E-02	14

(c) 2000-2014 Ingenuity Systems, Inc. All rights reserved.

Top Tox Functions

Assays: Clinical Chemistry and Hematology

Name	p-value	# Molecules
Increased Levels of ALT	1.32E-01 - 1.32E-01	2
Decreased Levels of Albumin	1.57E-01 - 2.64E-01	2
Decreased Levels of Hematocrit	2.39E-01 - 2.39E-01	1
Increased Levels of Hematocrit	3.49E-01 - 3.49E-01	4
Increased Levels of AST	3.80E-01 - 3.80E-01	1

Cardiotoxicity

Name	p-value	# Molecules
Cardiac Necrosis/Cell Death	3.29E-03 - 4.10E-01	10
Cardiac Hypertrophy	1.92E-02 - 1.00E00	13
Cardiac Arrythmia	3.35E-02 - 5.74E-01	9
Cardiac Enlargement	3.35E-02 - 3.35E-02	1
Cardiac Inflammation	3.35E-02 - 4.40E-01	5

Hepatotoxicity

Name	p-value	# Molecules
Liver Enlargement	1.12E-03 - 1.12E-03	2
Liver Necrosis/Cell Death	1.12E-03 - 4.90E-01	9
Hepatocellular Carcinoma	2.62E-03 - 1.91E-01	18
Liver Hyperplasia/Hyperproliferation	2.62E-03 - 6.02E-01	23
Liver Proliferation	2.10E-02 - 4.70E-01	8

Nephrotoxicity

(c) 2000-2014 Ingenuity Systems, Inc. All rights reserved.

Name	p-value	# Molecules
Renal Necrosis/Cell Death	1.77E-05 - 5.88E-01	19
Renal Damage	2.43E-02 - 6.15E-01	6
Nephrosis	3.35E-02 - 1.23E-01	4
Renal Inflammation	3.35E-02 - 1.00E00	5
Renal Nephritis	3.35E-02 - 1.00E00	5

Top Regulator Effect Networks

Top Networks

ID	Associated Network Functions	Score
1	Gene Expression, Protein Synthesis, Small Molecule Biochemistry	51
2	Cellular Compromise, DNA Replication, Recombination, and Repair, Hereditary Disorder	41
3	Cellular Development, Hair and Skin Development and Function, Nervous System Development and Function	38
4	Drug Metabolism, Endocrine System Development and Function, Lipid Metabolism	36
5	Hematological System Development and Function, Inflammatory Response, Tissue Morphology	31

Top Tox Lists

Name	p-value	Ratio
Increases Liver Hyperplasia/Hyperproliferation	1.66E-03	9/91 (0.099)
Renal Ischemic Resistance Panel (Rat)	3.76E-03	3/10 (0.3)
Increases Heart Failure	6.65E-03	4/23 (0.174)
Increases Depolarization of Mitochondria and Mitochondrial Membrane	1.79E-02	3/17 (0.176)
Hepatic Cholestasis	3.49E-02	9/142 (0.063)

(c) 2000-2014 Ingenuity Systems, Inc. All rights reserved.

INGENUITY® SYSTEMS

Top My Lists

Name	p-value	Ratio

Top My Pathways

Name	p-value	Ratio

Top Molecules

This analysis has no expression values.

(c) 2000-2014 Ingenuity Systems, Inc. All rights reserved.

INGENUITY® SYSTEMS
