# Supplementary material for: Systems biology surveillance decrypts pathological transcriptome remodeling
Source: BMC Syst Biol. 2015 Jul 17;9:36. doi: 10.1186/s12918-015-0177-8 (PMC4504166; doi:10.1186/s12918-015-0177-8)
Supplement: Additional file 1: — Functional enrichment data. Clustering Data: Provided are signaling pathways and gene networks enriched in each cluster, as well as gene IDs for all transcripts identified in the UMatrix analysis. Gene Ontology Data: Summarization of over represented functional themes in down and up regulated sub-transcriptomes for each of the truncation variants. [file 12918_2015_177_MOESM1_ESM.zip › 9929599221407335_add9.pdf]

Interactive pathway analysis of complex 'omics data

Analysis Name: Cluster 9 - 2014-06-04 08:14 PM
Analysis Creation Date: 2014-06-04
Build version: 308606M
Content version: 18488943 (Release Date: 2014-03-23)

Analysis settings

View

Reference set: Mouse Genome 430 2.0 Array

Relationship to include: Direct and Indirect

Includes Endogenous Chemicals

Optional Analyses: My Pathways My List

Filter Summary:

Consider only relationships where

confidence = Experimentally Observed

Cutoff:

(c) 2000-2014 Ingenuity Systems, Inc. All rights reserved.

Top Canonical Pathways

Name	p-value	Ratio
p53 Signaling	1.02E-03	6/113 (0.053)
Asparagine Biosynthesis I	1.26E-02	1/8 (0.125)
Phosphatidylethanolamine Biosynthesis III	1.26E-02	1/5 (0.2)
CDP-diacylglycerol Biosynthesis I	1.49E-02	2/27 (0.074)
Phosphatidylglycerol Biosynthesis II (Non-plastidic)	1.9E-02	2/33 (0.061)

Top Upstream Regulators

Upstream Regulator	p-value of overlap	Predicted Activation State
KLF9	3.60E-04	
MYC	4.01E-04	
EIF2B1	4.39E-04	
fludarabine	8.85E-04	
ZBTB17	1.49E-03	

(c) 2000-2014 Ingenuity Systems, Inc. All rights reserved.

Top Diseases and Bio Functions

Diseases and Disorders

Name	p-value	# Molecules
Gastrointestinal Disease	1.54E-03 - 4.95E-02	8
Organismal Injury and Abnormalities	1.54E-03 - 4.95E-02	37
Cancer	2.90E-03 - 4.95E-02	110
Endocrine System Disorders	2.90E-03 - 3.74E-02	8
Reproductive System Disease	2.90E-03 - 4.88E-02	23

Molecular and Cellular Functions

Name	p-value	# Molecules
Cellular Assembly and Organization	6.65E-05 - 4.95E-02	26
Cellular Function and Maintenance	4.81E-04 - 4.95E-02	26
Cellular Compromise	9.33E-04 - 3.92E-02	10
Lipid Metabolism	1.43E-03 - 4.95E-02	22
Molecular Transport	1.43E-03 - 4.95E-02	21

Physiological System Development and Function

Name	p-value	# Molecules
Embryonic Development	5.14E-04 - 4.95E-02	24
Hair and Skin Development and Function	5.14E-04 - 3.74E-02	6
Organ Development	5.14E-04 - 4.95E-02	18
Organismal Development	5.14E-04 - 4.95E-02	28
Tissue Development	5.14E-04 - 4.95E-02	23

(c) 2000-2014 Ingenuity Systems, Inc. All rights reserved.

Top Tox Functions

Assays: Clinical Chemistry and Hematology

Name	p-value	# Molecules
Increased Levels of Red Blood Cells	5.59E-03 - 5.59E-03	5
Increased Levels of Albumin	8.50E-02 - 8.50E-02	1
Increased Levels of Hematocrit	3.10E-01 - 3.10E-01	2

Cardiotoxicity

Name	p-value	# Molecules
Cardiac Arteriopathy	1.26E-02 - 4.47E-01	4
Cardiac Dilation	3.74E-02 - 3.74E-02	1
Cardiac Damage	9.66E-02 - 9.66E-02	1
Cardiac Inflammation	1.94E-01 - 1.94E-01	1
Cardiac Hypertrophy	2.72E-01 - 1.00E00	2

Hepatotoxicity

Name	p-value	# Molecules
Liver Necrosis/Cell Death	3.39E-03 - 3.26E-01	8
Liver Dysplasia	7.33E-02 - 7.33E-02	1
Liver Hematopoiesis	8.50E-02 - 8.50E-02	1
Liver Damage	1.90E-01 - 2.53E-01	3
Liver Proliferation	2.04E-01 - 3.11E-01	4

Nephrotoxicity

Name	p-value	# Molecules
Renal Hypertrophy	1.13E-02 - 2.53E-01	3
Nephrosis	1.26E-02 - 1.26E-02	1

(c) 2000-2014 Ingenuity Systems, Inc. All rights reserved.

INGENUITY SYSTEMS

Renal Necrosis/Cell Death	8.50E-02 - 3.85E-01	8
Glomerular Injury	2.04E-01 - 4.61E-01	2
Renal Hyperplasia/Hyperproliferation	2.04E-01 - 2.04E-01	1

Top Regulator Effect Networks

Top Networks

ID	Associated Network Functions	Score
1	Cardiovascular System Development and Function, Cell Morphology, Cell-To-Cell Signaling and Interaction	47
2	Embryonic Development, Hair and Skin Development and Function, Organ Development	44
3	Cancer, Digestive System Development and Function, Cellular Development	36
4	Embryonic Development, Organ Development, Organ Morphology	26
5	Cell Cycle, Cellular Movement, Developmental Disorder	24

Top Tox Lists

Name	p-value	Ratio
p53 Signaling	1.08E-03	6/99 (0.061)
Liver Necrosis/Cell Death	1.74E-02	8/271 (0.03)
CAR/RXR Activation	3.36E-02	2/29 (0.069)
FXR/RXR Activation	8.26E-02	3/86 (0.035)
Genes Downregulated in Response to Chronic Renal Failure (Rat)	8.5E-02	1/10 (0.1)

(c) 2000-2014 Ingenuity Systems, Inc. All rights reserved.

Top My Lists

Name	p-value	Ratio

Top My Pathways

Name	p-value	Ratio

Top Molecules

This analysis has no expression values.

(c) 2000-2014 Ingenuity Systems, Inc. All rights reserved.
