# Supplementary figures and images for: Systems biology surveillance decrypts pathological transcriptome remodeling
Source: BMC Syst Biol. 2015 Jul 17;9:36. doi: 10.1186/s12918-015-0177-8 (PMC4504166; doi:10.1186/s12918-015-0177-8)

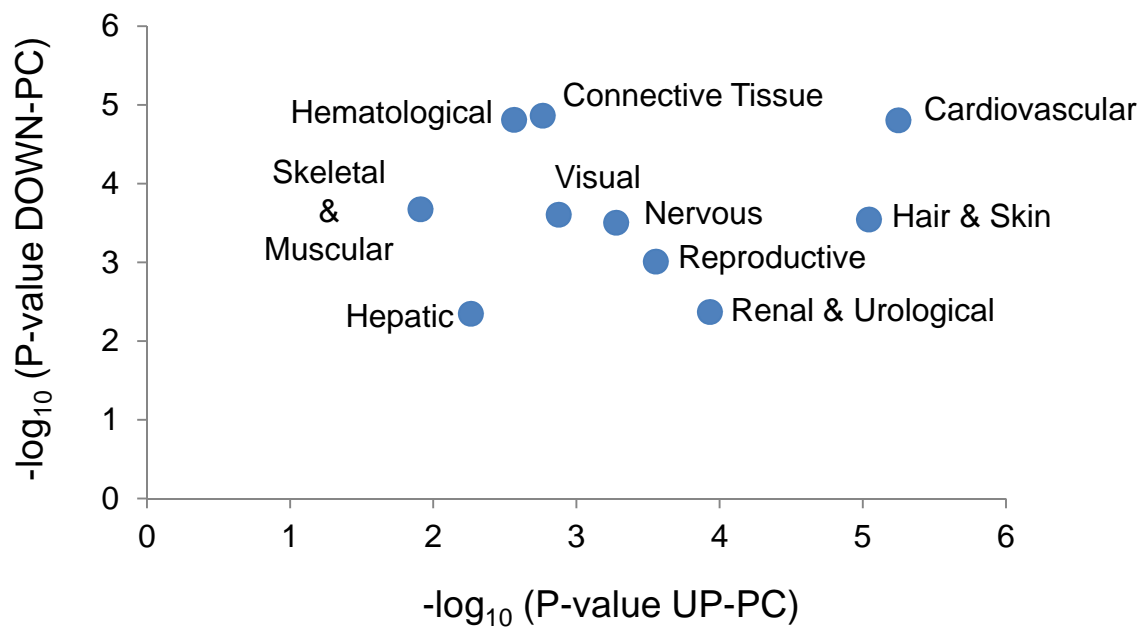

Supplemental Figure 1

Supplement: Additional file 3: Figure S1. — Physiological systems impacted by CALR-/--PC. Extraction of physiological development categories enriched in the CALR-/--PC transcriptome revealed prioritization of Cardiovascular System Development and Function, independent of up or down regulated gene expression trends. Enrichment P-values for each physiological category in the up and down regulated sub-transcriptome cohorts were transformed into their –log values and plotted for ease of visualization. [file 12918_2015_177_MOESM3_ESM.pdf]
